# Phosphorylation of USP29 by CDK1 Governs TWIST1 Stability and Oncogenic Functions

**DOI:** 10.1002/advs.202205873

**Published:** 2023-02-13

**Authors:** Tangming Guan, Mei Li, Yan Song, Jiayi Chen, Jiaxin Tang, Caishi Zhang, Yalei Wen, Xiao Yang, Lei Huang, Yingjie Zhu, Hongxian Wang, Ke Ding, Junxia Zheng, Haoxing Zhang, Tongzheng Liu

**Affiliations:** ^1^ College of Pharmacy/International Cooperative Laboratory of Traditional Chinese Medicine Modernization and Innovative Drug Development of Ministry of Education (MOE) of China Jinan University Guangzhou 510632 China; ^2^ Department of Pathology National Cancer Center/National Clinical Research Center for Cancer/Cancer Hospital Chinese Academy of Medical Sciences and Peking Union Medical College Beijing 100021 China; ^3^ Guangdong Provincial Key Laboratory of Genome Stability and Disease Prevention College of Life Sciences and Oceanography Shenzhen University Shenzhen 518055 China; ^4^ Department of Thyroid and Breast Surgery Shenzhen Nanshan People's Hospital & The 6th Affiliated Hospital of Shenzhen University Shenzhen 518052 China; ^5^ State Key Laboratory of Bioorganic and Nature Product Chemistry Shanghai Institute of organic chemistry Shanghai 200032 China; ^6^ School of Biomedical and Pharmaceutical Sciences Guangdong University of Technology Guangzhou 510006 China

**Keywords:** CDK1, chemotherapeutic resistance, metastasis, TWIST1, USP29

## Abstract

Triple‐negative breast cancer (TNBC) is a highly lethal malignancy with limited therapy options. TWIST1, a key transcriptional factor of epithelial‐mesenchymal transition (EMT), contributes to self‐renewal of cancer stem‐like cells (CSCs), chemo‐resistance, metastasis, and TNBC‐related death. However, the mechanism by which TWIST1 is deregulated in TNBC remains elusive. Here, USP29 is identified as a bona fide deubiquitinase of TWIST1. The deubiquitination of TWIST1 catalyzed by USP29 is required for its stabilization and subsequent EMT and CSC functions in TNBC, thereby conferring chemotherapeutic resistance and metastasis. Furthermore, the results unexpectedly reveal that CDK1 functions as the direct USP29 activator. Mechanistically, CDK1‐mediated phosphorylation of USP29 is essential for its deubiquitinase activity toward TWIST1 and TWIST1 driven‐malignant phenotypes in TNBC, which could be markedly mitigated by the genetic ablation or pharmacological inhibition of CDK1. Moreover, the histological analyses show that CDK1 and USP29 are highly upregulated in TNBC samples, which positively correlate with the expression of TWIST1. Taken together, the findings reveal a previously unrecognized tumor‐promoting function and clinical significance of the CDK1‐USP29 axis through stabilizing TWIST1 and provide the preclinical evidence that targeting this axis is an appealing therapeutic strategy to conquer chemo‐resistance and metastasis in TNBC.

## Graphical abstract

In this study, USP29 is revealed as a bona fide deubiquitinase of TWIST1, a key transcriptional factor of epithelial‐mesenchymal transition and contributes to stemness, chemo‐resistance and metastasis of triple‐negative breast cancer (TNBC). Furthermore, CDK1‐mediated phosphorylation of USP29 governs TWIST1 stability and oncogenic functions. Targeting CDK1‐USP29‐TWIST axis is an appealing therapeutic strategy to conquer chemo‐resistance and metastasis in TNBC.

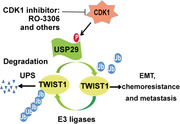

## 1 Introduction

Triple‐negative breast cancer (TNBC) is the subtype of breast cancer with negative expression of estrogen (ER), progesterone (PR), and human epidermal growth factor receptor‐2 (HER2) and account for ≈10% of all breast cancers.^[^ ^1^ ^]^ Due to the specific molecular expression pattern, TNBC has no targeted therapy and chemotherapy such as cisplatin and paclitaxel is still the standard treatment strategies.^[^ ^2^ ^]^ However, the efficacy of chemotherapy is unsatisfactory due to the frequent chemo‐resistance and metastasis, which makes TNBC the most lethal subtype among all breast cancer.^[^ ^3^ ^]^ Therefore, to elucidate key molecular mechanisms that specially contribute to cancer metastasis and chemo‐resistance would be crucial for the discovery of new therapeutic targets and greatly benefit the clinical outcome of patients with TNBC.

The epithelial‐mesenchymal transition (EMT) is the reversible reprogramming process of differentiated epithelial cells to a mesenchymal‐like phenotype. EMT is originally identified as a critical process in embryonic development and could also be triggered by the underlying transcription control factors, so‐called EMT‐TFs, in response to deregulated network of cytokines/growth factors in tumor microenvironment during tumor progression/metastasis.^[^ ^4^ ^]^ Accumulating evidence showed that EMT and its EMT‐TFs are of importance for the acquisition of cancer stem‐like cell (CSC) properties, which induce cancer development, progression, and metastasis due to their capacity to be chemo‐resistant, invasive, and dormant.^[^ ^4^, ^5^ ^]^ TWIST1, as a key EMT‐TF, is shown to drive EMT, CSC self‐renewal, and metastasis of breast cancer, and desensitize cancer cells to chemotherapy.^[^ ^6^ ^]^ More importantly, the aberrant upregulation of TWIST1 was frequently observed in tumor samples from TNBC and high‐grade breast cancer compared to the low expression in normal human mammary tissues, which was correlated with tumor stage, metastasis, and poor clinical outcome of patients with TNBC_._^[^ ^6^, ^7^ ^]^ Concerning the unequivocally pathological significance of TWIST1 in EMT, CSCs, and cancer progression, specifically targeting TWIST1 might be a compelling approach in the management of TNBC.

The expression of TWIST1 could be tightly regulated at the transcriptional and post‐transcriptional levels. Several cytokines and growth factors such as IL‐6, TGF‐*β*, WNT, and EGF presented in the tumor microenvironment were reported to increase the expression of TWIST1 transcriptionally, which could repress E‐cadherin expression and then induce EMT in cancer cells.^[^ ^4^, ^8^ ^]^ On the other hand, the protein turnover and activation of TWIST1 could be regulated by the post‐translational modification of ubiquitin. Several E3‐ubiquitin ligases such as FBXL14, *β*‐TrCP, and RNF8 catalyze the ubiquitination of TWIST1 and promote its degradation or activation.^[^ ^6^, ^9^ ^]^ However, how TWIST1 is stabilized in TNBC remains elusive. Thus, to dissect the mechanism controlling TWIST1 stability will provide important clues for potential therapeutic strategy to conquer chemo‐resistance and cancer metastasis in TNBC.

Here, we identify USP29 as a bona fide deubiquitinating enzyme (DUB) of TWIST1. USP29 directly binds, deubiquitinates, and stabilizes TWIST1, which in turn promotes TWIST1‐driven tumor progression in TNBC. Furthermore, we unexpectedly identify that cyclin‐dependent kinase 1 (CDK1) functions as a direct activator of USP29. CDK1‐mediated phosphorylation is essential to activate USP29 toward TWIST1 and promote tumor progression in TWIST1 dependent manner. More importantly, the genetic ablation or the pharmacological inhibition of CDK1 could deactivate USP29 and subsequently destabilize TWIST1, thereby largely mitigating EMT, CSC self‐renewal, and metastasis, and sensitizing TNBC cells to chemotherapy. Overall, our study reveals that the CDK1‐USP29‐TWIST1 axis functions as an important regulatory mechanism of EMT, CSC self‐renewal, chemo‐resistance, and metastasis, which provides novel targeted strategies for the treatment of TNBC.

## 2 Results

### 2.1 Identification of USP29 as the Bona Fide Deubiquitinase of TWIST1

**Figure 1:**
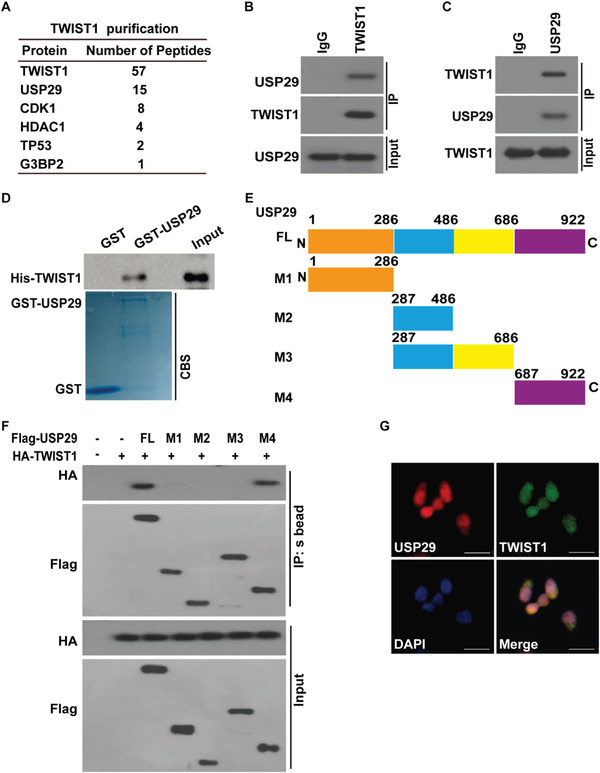
USP29 interacts with TWIST1. A) List of TWIST1‐associated proteins identified by mass spectrometric analysis. MDA‐MB‐231 cells stably expressing Flag‐TWIST1 were generated and TWIST1 complexes were subjected to mass spectrometric analysis. B,C) MDA‐MB‐231 cell lysates were subjected to immunoprecipitation with B) IgG, anti‐TWIST1 or C) anti‐USP29 antibodies. The immunoprecipitates were blotted with indicated antibodies. D) Purified recombinant GST, GST‐USP29 and His‐TWIST1 were incubated in vitro and the interaction between USP29 and TWIST1 was examined. CBS, Coomassie blue staining. E) Schematic showing the generation of the USP29 (full‐length (FL), M1 (1‐286aa), M2 (287‐486aa), M3 (287‐686aa) and M4 (687‐922aa)) constructs. F) Full length Flag‐USP29 or its truncation mutants were co‐expressed with HA‐TWIST1 in 293T cells. Cell lysates were subjected to immunoprecipitation with S‐protein Agaroses. The immunoprecipitates were blotted with indicated antibodies. G) Co‐localization of endogenous USP29 and TWIST1 in MDA‐MB231 cells was detected by immunofluorescence (IF) staining. Scale bars, 25 µm.

To elucidate potential mechanisms to stabilize TWIST1 in TNBC, we performed tandem affinity purification and mass spectrometry analysis using MDA‐MB‐231 cells stably expressing Flag‐TWIST1. We identified several potential TWIST1 interacting proteins including the DUB USP29 (**Figure** **1**A). We next confirmed the endogenous TWIST1‐USP29 interaction by co‐immunoprecipitation assay. As shown in Figure 1B,C and Figure S1A,B (Supporting Information), TWIST1 co‐immunoprecipitated with USP29 in MDA‐MB‐231, BT549, and HCC1806 cells, respectively. Reciprocal immunoprecipitation with USP29 antibodies also brought down TWIST1. In addition, purified GST‐USP29 but not GST pulled down His‐TWIST1 in vitro, indicating the direct interaction between USP29 and TWIST1 (Figure 1D). Further, a series of USP29 deletion mutants were generated and subjected to the Co‐IP assay to map the specific region within USP29 to bind TWIST1 (Figure 1E). As shown in Figure 1F, only the full‐length (FL) and M4 mutant (687‐922aa) region of USP29, but not other mutants, could interact with TWIST1. The result of immunofluorescence staining also showed the co‐localization of USP29 with TWIST1 in the nucleus of TNBC cells (Figure 1G).

### 2.2 USP29 Deubiquitinates and Stabilizes TWIST1

**Figure 2:**
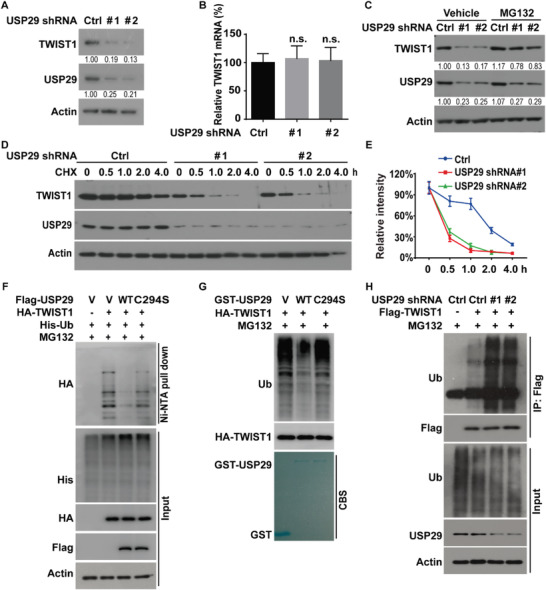
USP29 deubiquitinates and stabilizes TWIST1. A) MDA‐MB‐231 cells stably expressing control (Ctrl) or USP29 shRNAs (#1 and #2) were generated and western blot was performed with indicated antibodies. B) Total RNA was isolated from cells in (A). The expression of TWIST1 mRNA in cells was determined by quantitative PCR. Transcript levels were determined relative to GAPDH mRNA level and normalized relative to control. The results represent mean ± s.d. from three independent experiments. C) MDA‐MB‐231 cells stably expressing control or USP29 shRNAs were treated with vehicle or MG132 (10 µm) and western blot was performed with indicated antibodies. D) Cycloheximide pulse‐chase assay was performed in cells as in (A); E) the relative level of TWIST1 to *β*‐actin was measured by image J. The results represent mean ± s.d. from three independent experiments. F) Cells were cotransfected with indicated plasmids and Ni‐NTA bead was used to pull down His‐tagged ubiquitin, and the polyubiquitylated TWIST1 protein was examined by western blot. G) Cells were transfected with HA‐TWIST1 and treated with MG132 (10 µm) for 10 h. Cell lysates were immunoprecipitated with anti‐HA antibody and incubated with purified GST, GST‐USP29, or GST‐USP29 C294S mutant in a cell‐free condition. The polyubiquitylated TWIST1 protein was detected by anti‐ubiquitin antibody. H) MDA‐MB‐231 cells stably expressing control or USP29 shRNAs were transfected with indicated plasmids and treated with MG‐132 (10 µm) for 10 h. Cell lysates were subjected to immunoprecipitation with anti‐Flag antibody and the ubiquitination of TWIST1 protein was examined by western blot.

The direct interaction of USP29 and TWIST1 prompted us to examine a potential role of USP29 in the regulation of TWIST1 stability and function. First, USP29 and TWIST1 protein levels were examined in several luminal and basal like breast cancer cell lines. As shown in Figure S2A (Supporting Information), protein levels of USP29 and TWIST1 were much higher in basal like breast cancer cell lines. We next depleted USP29 with its specific short hairpin RNAs (shRNAs) in MDA‐MB‐231 and BT549 cells, respectively. As shown in **Figure** **2**A and Figure S2B (Supporting Information), the knockdown of USP29 significantly decreased the protein level of TWIST1. The regulation of TWIST1 by USP29 was not at the transcription level since no apparent difference in TWIST1 mRNA level was detected in cells stably expressing control and USP29 shRNAs (Figure 2B, Figure S2C, Supporting Information). Since USP29 was reported to regulate the stability of some substrates such as HIF1*α* and Snail1 in different cancer cells, we also examined the effect of USP29 on these proteins in TNBC. As shown in Figure S2D (Supporting Information), the depletion of USP29 in MDA‐MB‐231 and BT549 had no obvious effect of HIF1*α* when cells were cultured in nomoxia condition. Consistently to a previous report,^[^ ^22^ ^]^ the effect of USP29 on Snail1 is more significant in A549 and H1299 cells. However, the depletion of USP29 only slightly affected the expression of Snail1 in TNBC cells (Figure S2D,E, Supporting Information).

Moreover, the inhibition of proteasome‐mediated degradation by MG132 could rescue the decreased TWIST1 protein level in USP29‐deficient cells (Figure 2C, Figure S2F, Supporting Information), dictating that the regulation of TWIST1 by USP29 is mediated by the proteasome. In line with this observation, we found that TWIST1 protein was less stable in cells depleting USP29 assessed by cycloheximide pulse‐chase assay (Figure 2D,E). Meanwhile, the overexpression of USP29 wild type (WT), but not its catalytically inactive mutant C294S, dramatically increased the stability of TWIST1 in MDA‐MB‐231 and BT549 cells (Figure S2G,H, Supporting Information), suggesting that the stabilization of TWIST1 by USP29 in TNBC cells depends on its deubiquitinase activity.

Next, we performed the ubiquitination assay in different conditions. As shown in Figure 2F, USP29 WT but not its catalytically inactive mutant C294S could dramatically decrease the ubiquitination level of TWIST1. In addition, a significant decrease of polyubiquitinated TWIST1 was observed when incubated with purified GST‐WT USP29 rather than GST‐C294S mutant in vitro (Figure 2G). Consistently, the depletion of USP29 in MDA‐MB‐231 and BT549 cells markedly increased the ubiquitination level of TWIST1 (Figure 2H, Figure S2I, Supporting Information). These data reveal USP29‐mediated deubiquitination of TWIST1 as a critical mechanism controlling its stability. Next, the specific ubiquitin linkage of TWIST1 deubiquitinated by USP29 was examined. We found that TWIST1 could be ubiquitinated through both K48‐ and K63‐specific polyubiquitin chains (Figure S2J, Supporting Information), while USP29 catalyzed the cleavage of only K48, but not K63 polyubiquitin chain of TWIST1 (Figure S2K,L, Supporting Information). Taken together, these results suggest that USP29 is a bona fide DUB targeting TWIST1 protein for deubiquitination and stabilization.

### 2.3 USP29 Promotes Tumor Progression of TNBC through Stabilizing TWIST1

**Figure 3:**
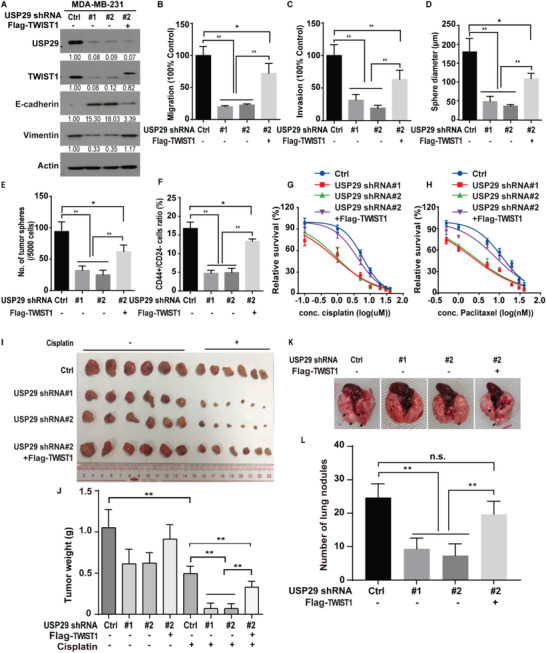
USP29 regulates EMT, CSC self‐renewal, metastasis, and cellular sensitivity to chemotherapy through TWIST1. A) MDA‐MB‐231 cells stably expressing control (Ctrl) or USP29 shRNAs (#1 and #2) were generated and western blot was performed with indicated antibodies. B,C) The migration and invasion abilities of cells as in (A) were measured by Transwell migration and invasion assays and results were quantified in (B,C). The results represent mean ± s.d. from three independent experiments; **p < 0.01. D,E) Graphic representation of mammosphere formation assay from cells described in (A). The results represent mean ± s.d. from three independent experiments; **p < 0.01. F) Graphic representation of the CD44^+^/CD24^−^ population from cells described in (A) was examined by FACS analysis. The results represent mean ± s.d. from three independent experiments; **p < 0.01. Cells as in (A) were treated with G) cisplatin or H) paclitaxel and cell survival was determined. The results represent mean ± s.d. from three independent experiments. I,J) Cells as in (A) were subcutaneously implanted into nude mice and mice were treated with saline or cisplatin (2 mg kg^−1^). I) Xenograft tumors were dissected and J) tumor weights were measured. The results represent the mean ± s.d. from six mice; **p < 0.01. K,L). Cells as in (A) were injected into the mammary fat pads of female NOD‐SCID mice. The primary tumors were surgically removed when tumor volume reached 400 mm^3^. After 8 weeks, mice were sacrificed and lung metastatic nodules were examined macroscopically. The results represent the mean ± s.d. from six mice; **P < 0.01.

Concerning the well‐established significance of TWIST1 in EMT, CSC self‐renewal, metastasis, and chemo‐resistance, we hypothesized that USP29 might promote these malignant processes through stabilizing TWIST1. We first overexpressed USP29 in luminal breast cancer cell MCF‐7, in which the expression of USP29 and TWIST1 is much lower than TNBC cells (Figure S2A, Supporting Information). As shown in Figure S3A,B (Supporting Information), the stable expression of USP29 decreased the expression of epithelial marker E‐cadherin and increased the expression of mesenchymal marker Vimentin. And the simultaneous depletion of TWIST1 largely reversed such an effect induced by USP29 overexpression (Figure S3A,B, Supporting Information), indicating the driven EMT process by USP29 is mediated by TWIST1. Of note, the depletion of USP29 in both MDA‐MB‐231 and BT549 cells significantly decreased the expression of TWIST1 and Vimentin, and increased the expression of epithelial marker E‐cadherin (**Figure** **3**A; Figure S3C,D, Supporting Information), while the reconstitution of TWIST1 in USP29‐deficient cells could dramatically rescue such a change (Figure 3A). Since USP29 regulates the stability of TWIST1 and consequently decreases the expression of E‐cadherin, we hypothesized that USP29 could be critical for the migration and invasion of TNBC cells in vitro. As expected, the silencing of USP29 significantly inhibited the migratory ability and invasiveness of MDA‐MB‐231 (Figure 3B,C) and BT549 cells (Figure S3G–I, Supporting Information) without affecting cell growth (Figure S3E,F, Supporting Information). And the reconstitution of TWIST1 largely restored the decreased migratory ability and invasiveness caused by USP29 deficiency (Figure 3B,C; Figure S3H,I, Supporting Information).

As TWIST1 is demonstrated to drive EMT and enhance CSC features,^[^ ^6^, ^8^, ^10^ ^]^ we next determined whether USP29 could regulate CSC self‐renewal in TNBC. Mammosphere formation assay, a well‐established assay to evaluate the self‐renewal ability of stem cells was performed. As shown in Figure 3D–F; Figure S3J–L (Supporting Information), the knockdown of USP29 in MDA‐MB‐231 and BT549 cells mitigated the size and number of mammosphere and the percentage of CD44^+^/CD24^−^ cell, a known marker of CSC subpopulation in breast cancer. The reconstitution of TWIST1 in USP29‐deficent cells markedly rescued such a change (Figure 3D–F; Figure S3J–L, Supporting Information). CSC with self‐renewing capacity is thought to be essential for tumor initiation.^[^ ^11^ ^]^ We next evaluated the effect of USP29 on the enrichment and activity of CSCs in vivo, limiting dilution assay (4 × 10^5^, 4 × 10^4^, or 1 × 10^4^ cells) of MDA‐MB‐231 cells stably expressing control or USP29 shRNA were subcutaneously injected into nude mice. In line with in vitro results, the depletion of USP29 significantly decreased the frequency of breast cancer stem cells (Figure S3M,N, Supporting Information). Collectively, these data demonstrate a crucial role of USP29 in promoting CSC features through TWIST1.

Emerging evidence suggests that EMT and CSCs critically contribute to cancer metastasis and resistance to chemotherapy.^[^ ^6^, ^12^ ^]^ We next examined the effect of USP29 on TNBC cellular sensitivity to currently used chemotherapies, such as cisplatin and paclitaxel. As shown in Figure 3G–J and Figure S3O–R (Supporting Information), the knockdown of USP29 significantly decreased the expression of TWIST1, and increased cancer cellular sensitivity to these chemotherapeutic agents both in vitro and in vivo, which could be largely reversed by the reconstitution of TWIST1 in USP29‐depleted cells. Since USP29 increases cancer cell migration and invasion through TWIST1 in vitro, we further investigated USP29 function in TNBC metastasis in vivo. As shown in Figure 3K,L, the depletion of USP29 in MDA‐MB‐231 cells markedly suppressed lung colonization, as determined by the number of metastatic lung nodules, whereas the reconstitution of TWIST1 could markedly rescue such an effect caused by USP29 deficiency. Together, these results uncover a previously unrecognized function of USP29 in tumor progression of TNBC through stabilizing TWIST1.

### 2.4 CDK1 Binds and Phosphorylates USP29

**Figure 4:**
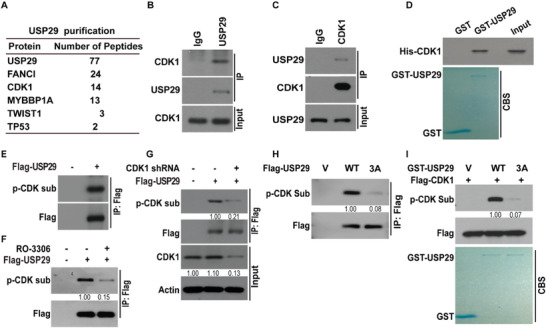
CDK1 binds and phosphorylates USP29. A) List of USP29‐associated proteins identified by mass spectrometric analysis. MDA‐MB‐231 cells stably expressing Flag‐USP29 were generated and USP29 complexes were subjected to mass spectrometric analysis. MDA‐MB‐231 cell lysates were subjected to immunoprecipitation with B) IgG, anti‐USP29 or C) anti‐CDK1 antibody. The immunoprecipitates were blotted with indicated antibodies. D) Purified recombinant GST, GST‐USP29 and His‐CDK1 were incubated in vitro as indicated. The interaction between USP29 and CDK1 was examined. CBS, Coomassie blue staining. E) Empty vector or Flag‐USP29 were transfected in cells stably expressing USP29 shRNA. Cell lysates were subjected to immunoprecipitation with anti‐Flag antibody and the phosphorylation of USP29 was examined by phospho‐CDK Substrate (p‐CDK sub) antibody. F) Cells were transfected with indicated plasmids and treated with vehicle, or CDK1 inhibitor (RO‐3306). Cell lysates were subjected to immunoprecipitation with anti‐Flag antibody and the phosphorylation of USP29 was examined by phospho‐CDK Substrate antibody. G) Cells were transfected with indicated plasmids. Cell lysates were subjected to immunoprecipitation with anti‐Flag antibody and the phosphorylation of USP29 was examined by phospho‐CDK Substrate antibody. H) Empty vector or Flag‐USP29 (WT or 3A mutant) was transfected in MDA‐MB‐231 cells stably expressing USP29 shRNA. Cell lysates were subjected to immunoprecipitation with anti‐Flag antibody and the phosphorylation of USP29 was examined by phospho‐CDK Substrate antibody. I) CDK1 phosphorylates USP29 in vitro. Cell lysates of MDA‐MB‐231 cells stably expressing Flag‐CDK1 were immunoprecipitated with anti‐Flag antibody and incubated with bacterial purified GST, GST‐USP29 WT, or GST‐USP29 3A mutant. Western blot was performed and the phosphorylation of USP29 was examined by phospho‐CDK Substrate antibody.

Our results demonstrate USP29 promotes tumor progression in TNBC through TWIST1. However, molecular mechanisms to regulate USP29 in TNBC remain elusive and strategies that directly target USP29 are not yet conceivable. Thus, we performed tandem affinity purification and mass spectrometry analysis in order to identify the potential regulators of the USP29‐TWIST1 axis. As shown in **Figure** **4**A, several proteins including the protein kinase CDK1 were identified as major USP29‐associated proteins. Co‐IP experiments were performed in both MDA‐MB‐231 and BT549 cells. As shown in Figure 4B,C and Figure S4A (Supporting Information), USP29 co‐immunoprecipitated with CDK1. Reciprocal immunoprecipitation with CDK1 antibodies also brought down USP29. In addition, purified GST‐USP29 but not GST could interact with His‐CDK1 in vitro, indicating the direct interaction between USP29 and CDK1 (Figure 4D). We also observed the interaction of USP29 with FANCI and MYBBP1A in HEK293 cells, two potential interactors of USP29 as shown in our mass spectrum results (Figure S4B, Supporting Information). However, the depletion of USP29 in MDA‐MB‐231 and BT‐549 cells did not significantly affect the expressions of these proteins (Figure S4C, Supporting Information). In addition, neither FANCI nor MYBBP1A depletion could affect the protein level of TWSIT1 in TNBC cells (Figure S4D,E, Supporting Information). These results indicate that FANCI or MYBBP1A might not be critically involved in the tumor‐promoting effect of USP29 in TNBC.

The overexpression of CDK1 has been detected in several types of human cancers and contributes to deregulated cell cycle, DNA damage response, protein synthesis, and other cell cycle independent functions.^[^ ^13^ ^]^ We next investigated whether CDK1 could phosphorylate USP29 and affect its tumor‐promoting function in TNBC. As shown in Figure 4E, Figure S4F (Supporting Information), the phosphorylation of USP29 was detected in both MDA‐MB‐231 and BT549 cells by using phospho‐CDK substrate antibody and the genetic ablation or the pharmacological inhibition of CDK1 by RO‐3306 could significantly reduce this phosphorylation event (Figure 4F,G, Figure S4G, Supporting Information). We next attempted to identify potential phosphorylation sites in USP29 by phosphorylation specific mass spectrometry analysis. We found several residues of USP29 could be phosphorylated, among which three residues Ser575, Thr578, and Ser672 matched with the consensus motif of CDK substrate (data not shown), which is highly conserved among primates (Figure S4H, Supporting Information). As shown in Figure 4H and Figure S4I,J (Supporting Information), the single mutation of USP29 S575A, T578A, or S672A only mildly decreased the phosphorylation of USP29, while the phosphorylation‐deficient 3A mutation (S575A/T578A/S672) almost completely abolished it. These results indicate that Ser575, Thr578, and Ser672 might be the major phosphorylation sites on USP29. To validate that CDK1 directly phosphorylates USP29, GST‐fused USP29 WT or the 3A mutant were incubated with active CDK1 and in vitro kinase assay was performed. As shown in Figure 4I, active CDK1 phosphorylated GST‐fused USP29 WT, was largely abolished by the 3A mutation. We also tested the possibility that CDK1 might act on TWIST1 directly. As shown in Figure S4K,L (Supporting Information), neither the interaction between CDK1 and TWIST1 nor the phosphorylation of TWIST1 could be detected. Collectively, these results suggest that CDK1 directly interacts with and phosphorylates USP29 other than TWIST1.

### 2.5 CDK1 Regulates TWIST1 Protein Stability, CSC Self‐Renewal, and Cellular Sensitivity to Chemotherapy

**Figure 5:**
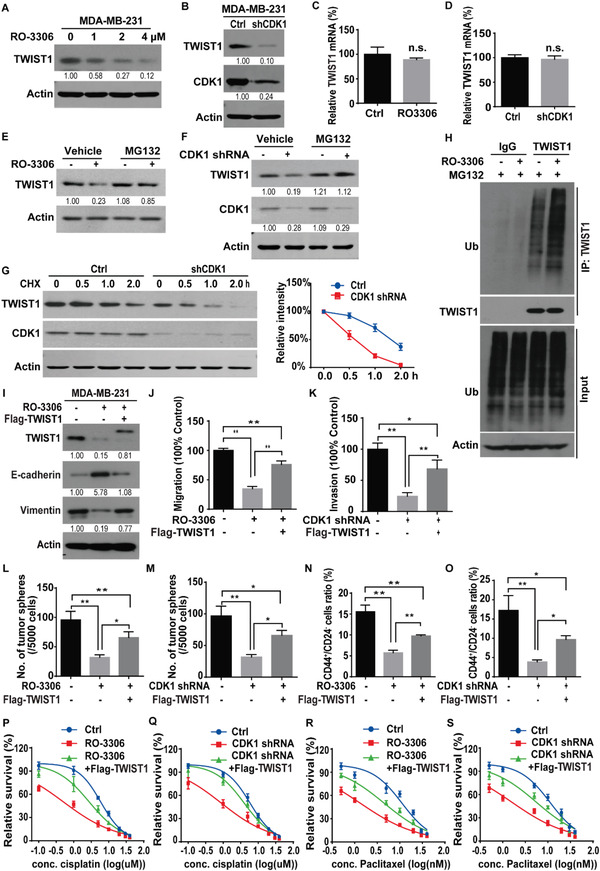
CDK1 regulates TWIST1 protein stability, CSC self‐renewal, and cellular sensitivity to chemotherapy. A) MDA‐MB‐231 cells were treated with indicated concentrations of RO‐3306 for 24 h and western blot was performed with indicated antibodies. B) Cells stably expressing control (Ctrl) or CDK1 shRNA were generated and western blot was performed with indicated antibodies. C,D) Total RNA was isolated from cells in (A) or (B). The expression of TWIST1 mRNA in cells was determined by quantitative PCR. Transcript levels were determined relative to GAPDH mRNA level and normalized relative to control. The results represent mean ± s.d. from three independent experiments. E) Cells were pretreated with vehicle or RO‐3306 for 24 h. Then, cells were treated with either vehicle or MG‐132 (10 µm) for an additional 6 h. TWIST1 level was detected by western blotting. F) Cells stably expressing control or CDK1 shRNA were treated with vehicle or MG132 and western blot was performed with indicated antibodies. G) Cycloheximide pulse‐chase assay was performed in cells as in (B) and results are quantified in right panel. The results represent mean ± s.d. from three independent experiments. H) Cells were treated with vehicle or RO‐3306 for 24 h in the presence of MG‐132 (10 µm) and cell lysates were subjected to immunoprecipitation with IgG or anti‐TWIST1. The polyubiquitylated TWIST1 protein was detected by anti‐ubiquitin antibody. I) MDA‐MB‐231 cells were transfected with indicated plasmids and treated with vehicle, or RO‐3306. Western blot was performed with indicated antibodies. J) Graphic representation of the migration ability from cells described in (I) was examined by Transwell migration assay. The results represent mean ± s.d. from three independent experiments; **p < 0.01. K) MDA‐MB‐231 cells stably expressing control or CDK1 shRNA were transfected with indicated plasmids. The invasiveness of cells was analyzed by Transwell invasion assay. The results represent mean ± s.d. from three independent experiments; **p < 0.01. L,M) Graphic representation of mammosphere formation assay from cells described in (I) or (K). The results represent mean ± s.d. from three independent experiments; *p < 0.05, **p < 0.01. N,O) Graphic representation of the CD44^+^/CD24^−^ population from cells described in (I) or (K) was examined by FACS analysis. The results represent mean ± s.d. from three independent experiments; *p < 0.05, **p < 0.01. Cells as in (I) or (K) were treated with P,Q) cisplatin or R,S) paclitaxel and cell survival was determined. The results represent mean ± s.d. from three independent experiments.

Regarding the fact that CDK1 phosphorylates USP29 and USP29 stabilizes TWIST1, we hypothesized that CDK1 might also affect TWIST1 stability and function in TNBC. As shown in **Figure** **5**A,B and Figure S5A,B (Supporting Information), the treatment of CDK1 inhibitor RO‐3306 or the depletion of CDK1 in MDA‐MB‐231 and BT549 cells significantly reduced the protein level of TWIST1 with no impact on its transcription (Figure 5C,D). The proteasome inhibitor MG132 could rescue such a decrease in TWIST1 protein level caused by the depletion or pharmacological inhibition of CDK1 (Figure 5E,F). We also found that TWIST1 protein was less stable in cells treated with RO‐3306 or with the depletion of CDK1 (Figure 5G; Figure S5C,D, Supporting Information), which could be due to the increased ubiquitination level of TWIST1 (Figure 5H; Figure S5E,F, Supporting Information).

Based on these results, we next examined whether CDK1 regulated TWIST1‐dependent malignant phenotypes. As shown in Figure 5I–K and Figure S5G–M,P,Q (Supporting Information), the genetic ablation or pharmacological inhibition of CDK1 significantly decreased the expression of TWIST1 and Vimentin, increased E‐cadherin expression, inhibited the migration and invasion ability of MDA‐MB‐231 and BT549 cells, which could be largely rescued by the reconstitution of TWIST1. Since TWIST1 critically contributes to breast CSC‐like properties, we also evaluated the function of CDK1 in CSC properties of breast cancer cells. We found that the depletion or inhibition of CDK1 in MDA‐MB‐231 and BT549 cells dramatically reduced the mammosphere formation efficiency (Figure 5L,M; Figure S5J,K,N,O,R,S, Supporting Information) and the percentage of CD44^+^/CD24^−^ cell (Figure 5N,O), while the reconstitution of TWIST1 could rescue such a defect (Figure 5L–O; Figure S5J,K,N,O,R,S, Supporting Information). In addition, the treatment of RO‐3306 or the depletion of CDK1 in MDA‐MB‐231 and BT549 cells significantly increased cellular sensitivity to cisplatin and paclitaxel, which could also be rescued by the reconstitution of TWIST1 (Figure 5P–S; Figure S5T–W, Supporting Information). Together, our results uncover a novel cell cycle independent function of CDK1 to promote tumor progression through stabilizing TWIST1 in TNBC.

### 2.6 CDK1‐Mediated Phosphorylation Activates USP29 toward TWIST1

**Figure 6:**
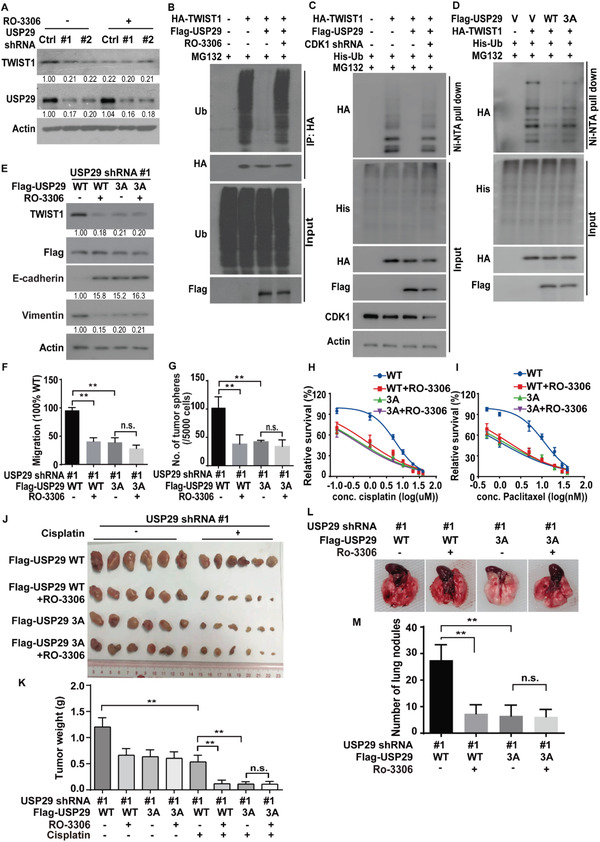
CDK1‐mediated phosphorylation of USP29 regulates TWIST1 protein stability and subsequent EMT, CSC self‐renewal, metastasis, and cellular sensitivity to chemotherapy. A) Cells stably expressing control or USP29 shRNA were transfected with indicated plasmids and treated with vehicle or RO‐3306 and western blot was performed with indicated antibodies. B) MDA‐MB‐231 cells were transfected with indicated plasmids and treated with vehicle or RO‐3306. Cell lysates were subjected to immunoprecipitation with anti‐HA antibody and the ubiquitination of TWIST1 protein was examined by western blot. C,D) Cells were cotransfected with indicated plasmids. Ni‐NTA bead was used to pull down His‐tagged ubiquitin and the polyubiquitylated TWIST1 protein was examined by western blot. E) MDA‐MB‐231 cells stably expressing USP29 shRNA were transfected with indicated plasmids and western blot was performed with indicated antibodies. F) Graphic representation of the migration ability from cells described in (E) was examined by Transwell migration assay. The results represent mean ± s.d. from three independent experiments; **p < 0.01. G) Graphic representation of mammosphere formation assay from cells described in (E). The results represent mean ± s.d. from three independent experiments; **p < 0.01. Cells as in (E) were treated with H) cisplatin or I) paclitaxel and cell survival was determined. The results represent mean ± s.d. from three independent experiments. J,K) Cells as in (E) were subcutaneously implanted into nude mice, and mice were treated with saline or cisplatin (2 mg kg^−1^). J) Xenograft tumors were dissected and K) tumor weights were measured. The results represent the mean ± s.d. from six mice; **p < 0.01. L,M) Cells as in (E) were injected into the mammary fat pads of female NOD‐SCID mice. The primary tumors were surgically removed when tumor volume reached 400 mm^3^. After 8 weeks, mice were sacrificed and lung metastatic nodules were examined macroscopically. The results represent mean ± s.d. from six mice; **P < 0.01.

Regarding the fact that both USP29 and CDK1 increase the stability of TWIST1, and CDK1 phosphorylates USP29, We hypothesized that the regulation of TWIST1 by CDK1 might be mediated through the activation of USP29 in TNBC. As shown in **Figure** **6**A, the pharmacological inhibition of CDK1 by RO‐3306 significantly decreased TWIST1 protein level in control cells, but could not cause any further reduction of TWIST1 levels in USP29‐deficent cells. In addition, the overexpression of USP29 dramatically decreased the ubiquitination level of TWIST1, which was markedly blocked by the treatment of RO‐3306 or the depletion of CDK1 (Figure 6B,C). These results suggest that CDK1 is critical for the enzymatic activity of USP29 toward TWIST1 in TNBC.

Phosphorylation could regulate protein‐protein interactions or the enzymatic activity of deubiquitinases.^[^ ^14^ ^]^ We next investigated whether the effect of CDK1 on TWIST1 and malignant processes in TNBC is mediated by the phosphorylation of USP29. We first examined whether CDK1 regulated the interaction between USP29 with TWIST1. As shown in Figure S6A (Supporting Information), the treatment of RO‐3306 in MDA‐MB‐231 cells did not affect the USP29‐TWIST1 interaction. We next examined whether CDK1‐mediated phosphorylation of USP29 could affect the ubiquitination level of TWIST1. As shown in Figure 6D and Figure S6B (Supporting Information), the overexpression of USP29 WT could dramatically decrease the ubiquitination level of TWIST1, whereas the 3A mutant failed to do so. In addition, we reconstituted shRNA‐resistant USP29 WT and 3A mutant in MDA‐MB‐231 and BT549 cells depleted of endogenous USP29. We found that the reduced TWIST1 protein level in USP29‐deficient cells was rescued by the reconstitution of USP29 WT, but not the 3A mutant (Figure S6C,D, Supporting Information). Likewise, the overexpression of USP29 WT in MDA‐MB‐231 and BT549 cells dramatically increased the half‐life of TWIST1 compared to the 3A mutant (Figure S6E,F, Supporting Information). These results dictate that CDK1‐mediated phosphorylation of USP29 is crucial for its deubiquitinase activity toward TWIST1.

We next examined the function of CDK1‐mediated phosphorylation of USP29 on TWIST1‐drivened malignant processes. As shown in Figure S6G,H (Supporting Information), the stable overexpression of USP29 in luminal breast cancer cell MCF‐7 decreased the level of epithelial marker E‐cadherin and increased the expression of mesenchymal marker Vimentin, whereas the 3A mutant failed to induce such phenotypes. We also found that the reconstitution of USP29 WT in endogenous USP29‐deficient MDA‐MB‐231 and BT549 cells significantly increased the migration and invasion ability, mammosphere formation efficiency, resistance to chemotherapy, as well as lung metastases compared to the 3A mutant (Figure 6E–M; Figure S6I–R, Supporting Information). Moreover, the pharmacological inhibition of CDK1 by RO‐3306 markedly decreased the migration (Figure 6F; Figure S6I,M, Supporting Information), invasion (Figure S6I,J,N, Supporting Information), the efficiency of mammosphere formation (Figure 6G; Figure S6I,K,O,P, Supporting Information), metastasis (Figure 6L,M) and sensitized cancer cells to chemotherapeutic drugs (Figure 6H–K; Figure S6Q,R, Supporting Information) in cells with the reconstitution of WT USP29. However, no obvious effects of RO‐3306 were observed in cells reconstituted with the 3A mutant (Figure 6E–M; Figure S6I–R, Supporting Information). These results indicate that CDK1‐mediated phosphorylation of USP29 is indispensable for its enzymatic activity toward TWIST1 as well as the TWIST1‐dependent malignant phenotypes in TNBC.

### 2.7 The Expression of CDK1 and USP29 Is Upregulated in TNBC and Is Positively Correlated with Poor Patient Outcomes

**Figure 7:**
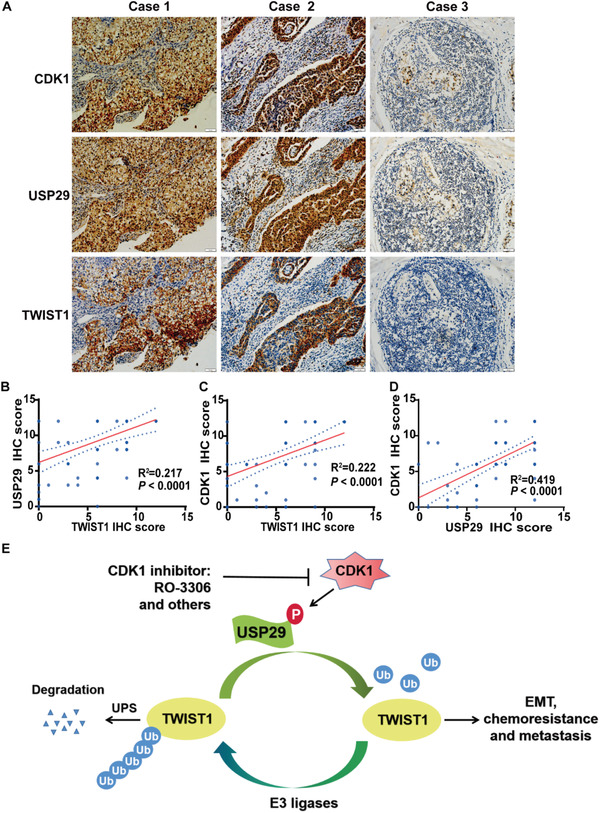
TWIST1 expression positively correlates with USP29 and CDK1 in TNBC. A) Representative images of immunohistochemical staining of CDK1, USP29, and TWIST1 in tumor samples of TNBC. Positive correlation of TWIST1 expression with B) USP29 and C) CDK1 was shown. D) Positive correlation of USP29 expression with CDK1 was shown. E) The working model to illustrate that CDK1 phosphorylation dependent activation of USP29 regulates EMT, chemo‐resistance, and metastasis through TWIST1.

To further examine the clinical relevance of the CDK1‐UPS29‐TWIST1 axis, we first analyzed the protein expression of CDK1, USP29, and TWIST1 in TNBC samples by immunohistochemistry. We found that the expression of USP29 was positively correlated with TWIST1 expression in TNBC (**Figure** **7**A,B; Figure S7A, Supporting Information). In addition, TWIST1 expression also positively correlated with CDK1 expression in TNBC (Figure 7A,C; Figure S7B, Supporting Information). Interestingly, through the analysis of public gene expression databases, we found that individuals with high USP29 expression had a significantly higher probability of developing relapse and distant metastasis in TNBC patients (Figure S7C,D, Supporting Information). Moreover, the high levels of CDK1 were correlated with poor relapse‐free survival (RFS) and distant metastasis‐free survival (DMFS) (Figure S7E,F, Supporting Information). Collectively, these results demonstrate that the dysregulated CDK1‐USP29‐TWIST1 axis contributes poor prognosis of TNBC.

## 3 Discussion and Conclusion

Despite advances in diagnosis and treatment in ER^+^ and HER2^+^ breast cancer, TNBC is still the most aggressive and lethal subtype with limited therapeutic options.^[^ ^3^, ^15^ ^]^ Resistance to conventional chemotherapy and cancer metastasis are the most common causes of death in patients with TNBC.^[^ ^3^, ^16^ ^]^ Therefore, it is urgent to develop new‐targeted therapies of TNBC.

The upregulation of TWIST1 in breast cancer cells and tissues was demonstrated to trigger EMT and enrich CSCs, which consequently contribute to cancer metastasis and chemo‐resistance.^[^ ^6^, ^12^ ^]^ Thus, targeting TWIST1 could be a promising strategy to conquer chemo‐resistance and metastasis of TNBC. However, TWIST1 is a short‐lived transcription factor lacking ligand‐binding domain, which renders directly targeting TWIST1 a challenge. To date, strategies to directly target TWIST1 are not conceivable yet. Therefore, upstream regulatory mechanisms to control the stability or function of TWIST1 protein might be a more druggable target for TWIST1 based therapy. In this study, we reveal several unexpected tumor‐promoting functions and the clinical significance of the CDK1‐USP29 axis through stabilizing TWIST1 and provide preclinical evidence demonstrating that targeting CDK1‐USP29 axis is an appealing therapeutic strategy to conquer chemo‐resistance and metastasis in TNBC (Figure 7E).

Although the role of TWIST1 is well established in chemotherapy resistance and metastasis, the mechanism to stabilize TWIST1 in TNBC remains largely unknown. Our study initially reveals the previously unrecognized function of USP29 in stabilizing TWIST1 and demonstrates its clinical relevance as a therapeutic target in TNBC. The evidence we observed is as follows. First, we identify USP29 as a bona fide deubiquitinase of TWIST1 through tandem affinity purification and mass spectrometry analysis. USP29 directly interacts with TWIST1, decreases its ubiquitination level through the DUB activity, thereby leading to the stabilization of TWIST1 (Figures 1 and 2; Figures S1 and S2, Supporting Information). Next, the genetic ablation of USP29 markedly attenuates TWIST1‐driven EMT, CSC self‐renewal, metastasis, and sensitizes cells to chemotherapies both in vitro and in vivo animal models (Figure 3 and Figure S3, Supporting Information). In addition, our results show that the reconstitution of TWIST1 could significantly rescue such phenotypes caused by the depletion of USP29 (Figure 3 and Figure S3, Supporting Information), implying that the tumor‐promoting activity of USP29 in TNBC largely depends on its ability to stabilize TWIST1. More importantly, our results reveal a tight clinical correlation between USP29 and TWIST1 in several breast cancer cell lines and human TNBC tumor specimens (Figure S2, Supporting Information and Figure 7). Both USP29 and TWIST1 are overexpressed in TNBC tumor specimens, but not in normal adult breast tissues, highlighting the potential of USP29 as a more druggable target toward TWIST1 stability in TNBC.

It is also noteworthy that we can not rule out the possibility of additional mechanisms engaged in UPS29‐regulated tumor progression of TNBC since the ectopic expression of TWIST1 could not completely rescue functional alterations caused by the depletion of USP29 in TNBC cells. Previously, USP29 was reported to regulate tumor metabolism and progression of neuroblastoma and B cell lymphoma by controlling MYC and HIF1*α* ubiquitination.^[^ ^17^ ^]^ In a cervical cancer study, the overexpression of USP29 enables the stabilization of the cell division cycle 25A (Cdc25A) and enhances the oncogenic potential of HeLa cells.^[^ ^18^ ^]^ In addition, USP29 was reported to promote gastric cancer cell migration by cooperating with phosphatase SCP1 to stabilize Snail protein.^[^ ^20^ ^]^ However, we found that the knockdown of USP29 significantly decreased the protein level of TWIST1, but only slightly affected the protein level of Snail1 in MDA‐MB‐231 and BT549 cells (Figure S2D, Supporting Information). This discrepancy might be due to the different cancer types or cell context. It would be necessary to investigate the potential involvements of other substrates of USP29 besides TWIST1 in TNBC metastasis and chemo‐resistance in the follow‐up studies.

Our study demonstrates that USP29 is a potential therapeutic target in the management of metastasis and chemo‐resistance in TNBC. Thus, it will be important to elucidate the mechanism by which the expression or activity of USP29 is upregulated in TNBC. To date, only a few studies reported that the expression of USP29 could be induced by different stimuli in various cancers. JTV1, one FBP partner, was shown to co‐activate FBP, induce USP29 transcription, and stabilize p53 in response to oxidative stress.^[^ ^19^ ^]^ In addition, the transcription of USP29 in gastric cancer cells could also be induced by TGF‐*β*, TNF*α*, and hypoxia, which were reported to be present in the tumor microenvironment and promote EMT and metastasis.^[^ ^20^ ^]^ However, the development of inhibitors to directly targeting TGF‐*β*, TNF*α*, hypoxia, or other stress could be a big challenge because of the complexity of downstream signaling pathways, difficulties in cell specificity, and uncontrolled toxicity. Thus, to identify regulatory mechanisms to regulate the activity of USP29 toward TWIST1 might be a more appropriate therapeutic strategy.

As one of the most post‐translational modification, phosphorylation has been widely implicated in the regulation of the activity, stability, subcellular localization of substrates. Intriguingly, several studies have revealed that the phosphorylation of deubiquitinases is important for their activity, substrate affinity, and ubiquitin recognition.^[^ ^14^ ^]^ We previously demonstrated that CDK4/6 binds and phosphorylates DUB3 at Ser41, which is essential for the activation of DUB3 and stabilization of Snail1, thereby promoting EMT and metastasis in breast cancer.^[^ ^21^ ^]^ This prompts us to investigate whether USP29 could be phosphorylated by certain protein kinases and whether this post‐translational modification is essential for its tumor‐promoting activity. Here, we reveal a previously uncharacterized function of CDK1 as the direct activator of USP29 in TNBC. CDK1, an important regulator of mitotic progression, was demonstrated to be aberrantly upregulated human malignant tumor tissues such as breast cancer, pancreatic cancer, melanoma, and CRC, which was positively correlated with pathological stage, lymphatic metastasis and poor clinical outcome of these cancers.^[^ ^13^, ^22^ ^]^ Recent study reported that high expression levels of CDK1 were correlated with the EMT‐related factors ZEB1 in human glioblastoma tissue specimens.^[^ ^23^ ^]^ In addition, the expression of CDK1 in prostate cancer cells could be regulated by TPX2, a microtubule‐associated protein that plays an important role in mitosis and cell cycle, thereby regulating the progress of EMT through the ERK/GSK3*β*/SNAIL pathway.^[^ ^24^ ^]^ However, the cell cycle independent mechanism of CDK1 to regulate tumor progression of TNBC remains poorly understood. Here, we initially reveal one cell cycle independent function of CDK1 to activate USP29, stabilize TWIST1 and promote TWIST1‐driven malignant processes, indicating the potential clinical implications of targeting CDK1 in the treatment of TNBC. First, we identify that CDK1 binds and phosphorylates USP29 at Ser575, Thr578, and Ser672, while the phosphorylation deficient 3A mutation almost abolishes the phosphorylation of USP29 (Figure 4 and Figure S4, Supporting Information). Second, either the genetic ablation or the pharmacological inhibition of CDK1 in TNBC cells significantly increases the ubiquitination and degradation of TWIST1, and consequentially blocks the TWIST1‐driven phenotypes in TNBC (Figure 5 and Figure S5, Supporting Information). Third, we demonstrate that CDK1 mediated phosphorylation is indispensable for the deubiquitinase activity of USP29 toward TWIST1 and TWIST1‐driven processes such as EMT, CSC self‐renewal, chemo‐resistance, and metastasis. The blocking of this phosphorylation event by targeting CDK1 or the reconstitution of the phosphorylation defective mutant USP229 3A fails to maintain such malignant features mentioned above (Figure 6 and Figure S6, Supporting Information). Ser575 and Thr578 are located in the ubiquitin carboxyl‐terminal hydrolase (UCH) domain (211‐600aa) of USP29, while Ser672 is outside of the UCH domain. All three sites are outside of the domain (687‐922aa) that mediates the interaction between USP29 and TWIST1. Although the phosphorylation sites on USP29 are not all located in the enzymatic region, phosphorylation may cause conformational changes of other non‐enzymatic region to affect substrate recognition and processing.^[^ ^25^ ^]^ Therefore, further efforts are warranted to investigate the structural of USP29 and the effect of these phosphorylation sites on the catalytic activity of USP29. More importantly, our histological analyses show that the upregulated expressions of CDK1 are highly correlated with TWIST1 in human TNBC specimens and poor patient survival in TNBC, indicating the clinical implication of CDK1 inhibitors in the treatment of cancers with high expression of USP29 and TWIST1 in TNBC (Figure 7 and Figure S7, Supporting Information). Although our study mostly focuses on TNBC, our finding that CDK1‐USP29‐TWIST1 axis affects CSC self‐renewal, cancer metastasis and the response to chemotherapeutic agents would have broader implications for the treatment of other cancers with high expression of USP29 and TWIST1 should be further examined in the future.

In conclusion, our study identifies the previously unknown oncogenic role of CDK1‐USP29 axis in regulating the stability of TWIST1 and demonstrates their clinical relevance as therapeutic targets in TNBC. Importantly, we provide preclinical evidence demonstrating that targeting CDK1‐USP29 axis might be effective to destabilize TWIST1 which decreases the CSCs population, tumor metastasis, and chemo‐resistance of TNBC. These would inspire the drug discovery and therapy development against TNBC.

## 4 Experimental Section

### Cell Culture, Plasmids, and Antibodies

HEK293T, HEK293, MDA‐MB‐231, BT549, HCC1806, MCF‐7, T47D cells, and other lines were obtained from ATCC (American Type Culture Collection). All cell lines were mycoplasma‐free and authenticated by short tandem repeat DNA profiling analysis. HEK293T, HEK293, MDA‐MB‐231, HCC1806, and MCF‐7 cells were cultured in DMEM (Gibco) medium supplemented with 10% FBS (ExCell Bio); BT549 and T47D cells were cultured in RPMI‐1640 (Gibco) medium supplemented with 10% FBS. All cells were maintained in a humidified cell incubator with 5% CO2 at 37 °C.

USP29, TWIST1, and CDK1 were cloned into pIRES‐EGFP, pLV.3‐Flag, pCMV‐HA, PET28A, and pGEX4T‐1 vectors. All site mutants were generated by site‐directed mutagenesis and identified by sequencing. USP29 shRNAs were purchased from Sigma‐Aldrich Co. The plasmid of TWIST1 shRNA was gifted from Dr. Jing Yang (University of California, San Diego, USA) and CDK1 shRNA was gifted from Dr. Bo Yang (Zhejiang University, China). The shRNA targeting sequences for CDK1 shRNA were 5′ ‐GCTGTACTTCGTCTTCTAATT A‐3′. The sequences for TWIST1 shRNA were 5′‐AAGCTGAGCAAGATTCAGACC‐3′. The sequences for USP29 shRNA#1 and #2 were 5′‐TGTGTGGAGTATCTTGGTGTA‐3′ and 5′‐CTGGTGAAGAATAACGAGCAA‐3′. The sequences for MYBBP1A shRNA#1 and #2 were 5′‐ATCTGCCTGAGACGCCTATTT‐3′ and 5′‐CTCAAAGCCGACTTGAATATA‐3′. The sequences for FANCI shRNA#1 and #2 were 5′‐CAATTGCCACGAACGGTAATG‐3′ and 5′‐ATGTAAGCTCGGAGCTAATAT‐3′.

Antibodies anti‐E‐cadherin (3195T, dilution: 1:1000), anti‐N‐cadherin (13116T, dilution: 1:1000), anti‐Vimentin (5741T, dilution: 1:1000) and anti‐CDK substrate antibody (9477S, dilution: 1:500) were purchased from CST (Cell Signaling Technology). Anti‐TWIST1 (ab50887, dilution: 1:1000) and anti‐MYBBP1A (ab99361, dilution: 1:1000) antibodies were purchased from Abcam. Anti‐CDK1 (19532‐1‐AP, dilution: 1:1000) anti FANCI (20789‐1‐AP, dilution: 1:1000) and anti‐cGAS (26416‐1‐AP, dilution: 1:1000) antibodies were purchased from Proteintech Group, Anti‐USP29 (sc‐517145, dilution: 1:1000) and anti‐Ub (sc‐8017) antibodies were purchased from Santa Cruz Biotechnology. Anti‐Flag (F1804, dilution: 1:1000), anti‐HA (H3663, dilution: 1:1000) and anti‐*β*‐actin (A1978, dilution: 1:5000) antibodies were purchased from Sigma‐Aldrich.

### Western Blotting Analysis

Cells were lysed in NETN buffer (pH 8.0, 300 mm NaCl, 20 mm Tris‐HCl, 0.5%NP‐40, 1 mm ethylenediaminetetraacetic acid (EDTA)) containing protease inhibitors (1× protease inhibitor cocktail (Roche), 1 mm sodium orthovanadate, 10 mm *β*‐glycerophosphate, 1 mm phenylmetnylsulfonyl fluoride, and 10 mm sodium fluoride). Proteins were separated by SDS‐PAGE gel electrophoresis and transferred to PVDF membranes, then incubated with the indicated primary and secondary antibodies.

### Immunoprecipitation

1) HEK293T cells transfected Flag‐USP29 and HA‐TWIST1 as indicated were lysed in NETN buffer and were incubated 4 h with Flag‐beads or HA‐beads for 4 °C. 2) MDA‐MB‐231 or BT549 cells as indicated were lysed in NETN buffer and were incubated overnight with primary antibodies together with protein A/G beads for 4 °C. The immunoprecipitates were subjected to western blotting after washing beads for three times.

### Denaturating Ni‐NTA Pulldown

Denaturating Ni‐NTA pulldown was performed as previously described.^[^ ^21^ ^]^

### Denaturing Immunoprecipitation for Ubiquitination

The cells were lysed in 100 mL 62.5 mm Tris‐HCl (PH 6.8), 10% glycerol, 2% SDS, 1 mm iodoacetamide, and 20 mm NEM, boiled for 15 min, diluted ten times with NETN buffer containing protease inhibitors, 20 mm NEM and 1 mm iodoacetamide, then centrifuged to remove cell debris. The cell extracts were subjected to immunoprecipitation with the indicated antibodies and blotted as indicated antibody.

### Immunofluorescence Staining

Cells were seeded onto glass coverslips for the experiment. Cells were washed with PBS, fixed with 4% formaldehyde for 20 min, permeabilized with 0.1% Triton X‐100 for 5 min, blocked with 0.5% BSA for 1 h, incubated with primary antibodies overnight at 4 °C and then incubated with secondary antibodies. The antibodies used in the immunofluorescence staining were E‐cadherin (3195T, 1:200; CST) and Vimentin (5741T, 1:200; CST). Localization of E‐cadherin and Vimentin was visualized by confocal microscopy.

### Glutathione S‐Transferase (GST) Pull‐Down Assay

Indicated cDNA was cloned into a pGEX‐4T‐1 vector with GST‐tag and purified from the Escherichia coli strain BL21 using Pierce Glutathione Agarose (Thermo Scientific). Bacterial‐expressed GST and GST‐USP29 protein bound to Pierce Glutathione Agarose was incubated with PET28A‐TWIST1, purified from the Escherichia coli strain BL21 overnight at 4 °C. Then the beads were washed with PBS four times, followed by western blotting.

### Flow Cytometry Analysis

Cells stably expressing Flag‐TWIST1 and CDK1 shRNA or CDK1 inhibitor RO‐3306 in MDA‐MB‐231 cells were digested with 0.25% trypsin at 37 °C. The cell pellets were collected by centrifugation (1500 rpm for 5 min) and then washed with PBS Twice. The cells were stained with antibodies anti‐CD44‐APC (BD Pharmingen, 559942) and anti‐CD24‐PE (BD Pharmingen, 555428) at 4 °C in the dark for 1 h. The pellets were then washed with PBS Twice and finally re‐suspended in 500 µL PBS. Flow cytometry analysis was performed with a FACS LSRFortessa flow cytometer.

### In Vitro Ubiquitination Assay

Cells transfected with HA‐TWIST1 were treated with 10 µm MG‐132 for 10 h. Proteins in the cell lysate were immunoprecipitated TWIST1 with HA‐beads, which was detected with an anti‐HA antibody. The recombinant GST‐tagged USP29 WT and C294S mutant protein were expressed in Escherichia coli strain BL21 and purified using Pierce Glutathione Agarose. The proteins were then eluted with GST washing buffer (10 mm GSH and 50 mm Tris‐HCl, pH = 8.0). The ubiquitinated TWIST1 protein was then incubated with purified GST‐USP29 WT and C294S protein separately for 4 h at 4 °C, followed by western blotting analysis.

### In Vitro Kinase Assay

Cells transfected with Flag‐CDK1 and collected after 48 h. Proteins in the cell lysate were immunoprecipitated CDK1 with Flag‐beads. The recombinant GST‐USP29 WT and 3A mutant protein were expressed in Escherichia coli strain BL21 and purified using Pierce Glutathione Agarose. The proteins were then eluted with GST washing buffer (10 mm GSH and 50 mm Tris‐HCl, pH = 8.0) and purified with Ultrafiltration tube. Purified CDK1 beads were finally incubated with purified GST‐USP29 WT and 3A in kinase buffer (50 mm Tris‐HCl, pH 7.4, 50 mm NaCl, 10 mm MgCl_2_, 10 mm *β*‐glycerophosphate, 1 mm dithiothreitol (DTT), and 100 µm ATP). The reaction was carried out at 30 °C for 30 min and stopped by the addition of a loading buffer. The samples were resolved by SDS‐PAGE, transferred onto PVDF, and followed by western blot analysis.

### Quantitative Real‐Time PCR (qRT‐PCR)

RNA extraction from cultured cells was performed using TRIzol reagent (Thermo Scientific, MA, USA), and then RNA was subsequently reverse transcribed to cDNA using a FastKing gDNA Dispelling RT SuperMix (Tiangen, Beijing, China). qRT‐PCR analysis was performed using FastFire qPCR PreMix (SYBR Green) using primers against TWIST1. All experiments were performed in triplicate with GAPDH as an internal control. All samples were normalized to GAPDH mRNA levels. Primer sequences were listed. hTWIST1 Forward: TAGATGTCATTGTTTCCAGAGAAGG, Reverse: ATTTCCAAGAAAATCTTTGGCA. GAPDH Forward: GATCGAATTAAACCTTATCGTCGT, Reverse: GCAGCAGAACTTCCACTCGGT.

### Migration and Invasion assay

For migration assays, cells were seeded in 24‐well Cell Culture Insert (BD, 353097). For invasion assays, cells were seeded in 24‐well Cell Culture Insert (BD, 353097) with matrigel. After 48 h, the filter was fixed with 4% Para‐formaldehyde and stained with 0.5% crystal violet, and then migrating and invading cells were counted.

### Cell Proliferation Assay

MDA‐MB‐231 or BT549 cells (4 × 10^4^) were seeded in 6‐well plates, and each group was in 6 wells. Cells for one of 6 wells were digested with 0.25% trypsin at 37 °C the next day. The cell pellets were collected by centrifugation (3000 rpm for 5 min), washed by PBS Twice, re‐suspended in PBS, and then counted in microscope. Likewise, cells for the next 5 days were counted in similar method.

### Tumor Sphere Formation Assay

The MDA‐MB‐231 and BT549 cells were harvested and suspended as single cells in stem‐cell culture medium, which was composed of DMEM/F12, supplemented with 20 ng mL^−1^ recombinant human EGF (rhEGF) and 10 ng mL^−1^ of basic fibroblast growth factor (bFGF) together with 1 × B27. After accurate cell counting, 5000 cells well^−1^ were added to a Low adhesion 24‐well plate contained 500 µL of stem‐cell culture medium, and each group was in 3 wells. A week later, images were taken with a fluorescence inverted microscope, and the spheres >50 cells were counted as the first generation. Then the cells were resuspended and counted, seeded 5000 cells well^−1^, and performed the above progress until the third generation.

### CCK‐8 Assay

A Cell Counting Kit‐8 (HY‐K0301) was used to measure proliferation of MDA‐MB‐231 and BT549 cells. A total of 2000 cells in a volume of 100 µL per well were cultured in four replicate wells in a 96‐well plate in medium containing 10% FBS. The cisplatin was treated with different concentrations for 72 h and CCK‐8 reagent (15 µL) was added and incubated for 2 h.

### Animal Studies

All animal experiments were performed in accordance with a protocol approved by the Institutional Animal Care and Use Committee of the Jinan University (2021125‐06). For subcutaneous xenografting, MDA‐MB‐231 and BT549 cells (1 × 10^6^) were subcutaneously implanted into female BALB/c nude mice (5–7 weeks old, n = 6) (Jicui Yaokang Biotechnology Co., Ltd., Jiangsu, China), respectively. The tumor volumes were measured three times weekly by using a Vernier caliper to measure the short diameter and long diameter of the tumor. Mice were administered saline or RO‐3306 (4 mg kg^−1^) every 2 days until sacrifice. Cisplatin (2 mg kg^−1^) was administered for three times weekly after xenografting. Tumor volumes were calculated using the following formula: width^2^ × length × 0.4 (mm^3^). After the tumors had grown for the designated time, all mice were euthanized, and the tumors were harvested. For the lung metastasis study, MDA‐MB‐231 cells (1 × 10^6^) were transfected as indicated and injected into the mammary fat pad of female NOD‐SCID mice (5–7 weeks old, n = 6) (Jicui Yaokang Biotechnology Co., Ltd., Jiangsu, China). When tumors reached 400 mm^3^ in size, the primary tumors were removed. Mice were sacrificed and the number of metastatic lung nodules was counted and quantified after 8 weeks.

### Immunohistochemical Staining

Tissue sections of TNBC were obtained from the tissue bank at the Shenzhen Nanshan People's Hospital & the 6th Affiliated Hospital of Shenzhen University in accordance with the approval document of the Institutional Medical Ethics Committee (0720001, ky‐2020‐039‐02). Anti‐TWIST1 (1:100; Proteintech Group; 25465‐1‐AP) and anti‐CDK1 (1:100; Proteintech Group; 19532‐1‐AP), anti‐USP29 (1:100; Santa Cruz, sc‐517145) antibodies were used for immunohistochemical staining of formalin‐fixed paraffin‐embedded of breast cancer tissues were incubated out at 4 °C for 12 h. The immunostaining was randomly scored by two pathologists. The IHC score was calculated by combining the quantity score (percentage of positively stained tissues) with the staining intensity score. The quantity score ranges from 0 to 4, For example, 0, no immunostaining; 1, 1–24% of tissues were stained; 2, 25–49% were positive; 3, 50–74% were positive; and 4, ≥ 75% of tissues were positive. The staining intensity was scored as 0 (negative), 1 (weak), 2 (moderate), and 3 (strong). The score for each tissue was calculated by multiplying the quantity with the intensity score (the range of this calculation was therefore 0–12). An IHC score of 9–12 was considered a strong immunoreactivity; 5–8, moderate; 1–4, weak; and 0, negative. Samples with IHC score > 4 were considered to be high, and ≤ 4 were considered to be low. The Fisher exact test was used for statistical analysis of the correlation between CDK1, USP29, and TWIST1.

### Statistics

For cell migration, invasion, and proliferation experiments, all data were analyzed by GraphPad Prism 9.3. Each experiment was independently performed three times, following the principle of repeatability. In the animal study, data represent the mean ± s.d. of six mice. The differences between the two groups of data were analyzed by *t*‐test, and the differences between multiple groups of data were compared using One/Two‐way ANOVA analysis of variance and Tukey's test: compare all pairs of columns. *P<0.05; **P<0.01. *P<0.05 was considered statistically significant.

## Conflict of Interest

The authors declare no conflict of interest.

## Author Contributions

T.G., M.L., and Y.S. contributed equally to this work. H.Z. and T.L. conceived the study and designed the experiments. T.G. and T.L. performed most of the experiments assisted by M.L., Y.S., J.C., J.T., C.Z., Y.W., X.Y., L.H., and Y.Z. T.G., M.L., and T.L. analyzed data and wrote the manuscript assisted by J.Z. and H.Z. All authors discussed the results and commented on the manuscript.

## Supporting information

Supporting information

## Data Availability

The data that support the findings of this study are available from the corresponding author upon reasonable request.
