# Supplementary material for: Phosphorylation of USP29 by CDK1 Governs TWIST1 Stability and Oncogenic Functions
Source: Adv Sci (Weinh). 2023 Feb 13;10(11):2205873. doi: 10.1002/advs.202205873 (PMC10104637; doi:10.1002/advs.202205873)

ADVANCED SCIENCE
Open Access

Supporting Information

for *Adv. Sci.*, DOI 10.1002/advs.202205873

Phosphorylation of USP29 by CDK1 Governs TWIST1 Stability and Oncogenic Functions

Tangming Guan, Mei Li, Yan Song, Jiayi Chen, Jiaxin Tang, Caishi Zhang, Yalei Wen, Xiao Yang, Lei Huang, Yingjie Zhu, Hongxian Wang, Ke Ding, Junxia Zheng*, Haoxing Zhang* and Tongzheng Liu**

Supplementary Figure Legends

Supplementary Figure 1. USP29 interacts with TWIST1.

A-B) Cell lysates of BT549 (A) and HCC1806 (B) were subjected to immunoprecipitation with IgG, anti-TWIST1 or anti-USP29 antibodies. The immunoprecipitates were blotted with indicated antibodies.

Supplementary Figure 2. USP29 deubiquitinates and stabilizes TWIST1.

A) Cell lysates of different luminal and basal like subtypes of human breast cancer cell lines were immunoblotted to examine the expression of TWIST1 and USP29. B) Cell lysates of BT549 cells stably expressing control (Ctrl) or USP29 shRNAs (#1 and #2) were immunoblotted with indicated antibodies. C) Total RNA was isolated from cells in (B). The expression of TWIST1 mRNA in cells was determined by quantitative PCR. Transcript levels were determined relative to GAPDH mRNA level and normalized relative to control. The results were represented as mean ± s.d. from three independent experiments. D-E) Cell lysates of MDA-MB-231 and BT549 (D), A549 and H1299 (E) cells stably expressing control (Ctrl) or USP29 shRNAs (#1 and #2) were immunoblotted with indicated antibodies. F) Cell lysates of BT549 cells stably expressing control or USP29 shRNAs were treated with vehicle or MG132 immunoblotted with indicated antibodies. G-H) MDA-MB-231 (G) and BT549 (H) Cells were transfected with empty vector, USP29 WT or USP29 C294S mutant followed with cycloheximide pulse-chase assay. The results are quantified in lower panel. The results were represented as mean ± s.d. from three independent experiments. I) BT549 cells stably expressing control or USP29 shRNAs were transfected with indicated plasmids and treated with MG-132 for 10 hours. Cell lysates were subjected to immunoprecipitation with anti-Flag antibody and the ubiquitination of TWIST1 protein was examined by western blot. J) Identification of the type of ubiquitin linkage on TWIST1. Flag-TWIST1 and HA-tagged ubiquitin were co-transfected and the cell lysates were immunoprecipitated with anti-Flag antibody and immunoblotted as indicated. K-L) USP29 WT, not the C294S mutant, remove the K48 ubiquitination of TWIST1 (K), but has no effect on TWIST1 K63

ubiquitination (L). Cells were transfected with indicated plasmids. Cell lysates were immunoprecipitated with s-protein agaroses and immunoblotted as indicated.

Supplementary Figure 3. USP29 regulates EMT, CSC self-renewal and cellular sensitivity to chemotherapy through TWIST1.

A) MCF-7 cells were transfected with indicated plasmids and immunoblotted with indicated antibodies. B) Immunofluorescence (IF) images of E-cadherin and Vimentin in MCF-7 cells described in (A). Nuclei were immunostained wtih DAPI (blue). Scale bars=25 μm. C-D) MDA-MB-231 (C) and BT549 (D) Cell lysates of cells stably expressing control (Ctrl) or USP29 shRNAs (#1 and #2) were immunoblotted with indicated antibodies. E-F) Cells as in (C-D) were seeded in each well (4x104/well) and cell number was counted every 24 hours. The results were represented as mean ± s.d. from three independent experiments. G) BT549 cells stably expressing control (Ctrl) or USP29 shRNAs (#1 and #2) were transfected with indicated plasmids and immunoblotted with indicated antibodies. H-I) Graphic representation of the migration (H) and invasion (I) capacity of cells described in (G) were examined by migration and invasion assays. The results were represented as mean ± s.d. from three independent experiments; $^{**}p<0.01$. J-K) Graphic representation of mammosphere formation assay from cells described in (G). The results represent mean ± s.d. from three independent experiments; $^{**}p<0.01$. L) Graphic representation of the $CD44^{+}/CD24^{-}$ population from cells described in (G) was examined by FACS analysis. The results represent mean ± s.d. from three independent experiments; $^{**}p<0.01$. M, N) MDA-MB-231 cells as in (C) were subcutaneously injected ($4x10^5$, $4x10^4$ or $1x10^4$ cells per mouse) into nude mice. Tumor formation ability and stem cell frequency were analyzed. O) MDA-MB-231 cells stably expressing control (Ctrl) or USP29 shRNAs (#1 and #2) were subcutaneously implanted into nude mice. Xenograft tumors were extracted 6 weeks later and western blotting was performed with anti-TWIST1 antibody. P) Cells as in (G) were treated with cisplatin or paclitaxel and cell survival was determined. The results represent mean ± s.d. from three independent experiments. Q-R) BT549 Cells as in (G) were subcutaneously implanted

into nude mice and mice were treated with saline or cisplatin (2 mg/kg). Xenograft tumors were dissected (Q) and tumor weights were measured (R). The results represent the mean ± s.d. from six mice; ** $p < 0.01$, * $p < 0.05$.

Supplementary Figure 4. CDK1 binds and phosphorylates USP29.

A) BT549 cell lysates were subjected to immunoprecipitation with IgG, anti- USP29 or anti-CDK1 antibodies followed with immunoblot with indicated antibodies. B) Empty vector or Flag-USP29 were transfected in HEK 293 cells and cell lysates were subjected to immunoprecipitation with IgG, or anti-Flag antibodies. The immunoprecipitates were blotted with indicated antibodies. C) Cell lysates of MDA-MB-231 and BT549 cells stably expressing control or USP29 shRNAs immunoblotted with indicated antibodies. D-E) Cell lysates of MDA-MB-231 and BT549 cells stably expressing control, MYBBP1A shRNAs or FANCI shRNAs immunoblotted with indicated antibodies. F) Empty vector or Flag-USP29 were transfected in BT549 cells. Cell lysates were subjected to immunoprecipitation with anti-Flag antibody and the phosphorylation of USP29 were examined by phospho-CDK Substrate (p-CDK sub) antibody. G) BT549 cells were transfected with indicated plasmids and treated with vehicle or CDK1 inhibitor (RO-3306). Cell lysates were subjected to immunoprecipitation with anti-Flag antibody and the phosphorylation of USP29 were examined by phospho-CDK Substrate antibody. H) Sequence conservation analysis of the amino acids sequences flanking around S575, T578 and S672 residue of USP29 are across different species. Arrows: Serine and Threonine residues remain conserved across species. I) Cells stably expressing USP29 shRNA were transfected with indicated plasmids. Cell lysates were subjected to immunoprecipitation with anti-Flag antibody the phosphorylation of TWIST1 were examined by phospho-CDK substrate antibody. J) Empty vector or Flag-USP29 (WT or 3A mutant) were transfected in BT549 cells stably expressing USP29 shRNA. Cell lysates were subjected to immunoprecipitation with anti-Flag antibody and the phosphorylation of USP29 were examined by phospho-CDK substrate antibody. K) MDA-MB231 cells were transfected with empty vector or Flag-TWIST1. Cell lysates

were subjected to immunoprecipitation with anti-Flag antibody and immunoblotted with indicated antibodies. L) MDA-MB-231 cells were transfected with empty vector or Flag-TWIST1. Cell lysates were subjected to immunoprecipitation with anti-Flag antibody and the phosphorylation of TWIST1 were examined by phospho-CDK substrate antibody.

Supplementary Figure 5. CDK1 regulates TWIST1 protein stability and CSC self-renewal.

A) BT549 cells were treated with indicated concentrations of RO-3306 for 24 hours and immunoblotted with indicated antibodies. B) BT549 cells stably expressing control (Ctrl) or CDK1 shRNA were immunoblotted with indicated antibodies. C) MDA-MB-231 cells were pretreated with RO-3306 (2 μM) for 24 hours and followed with cycloheximide pulse-chase assay. Results are quantified and represented as mean ± s.d. from three independent experiments (Right panel). D) BT549 cells were pretreated with RO-3306 (2 μM) for 24 hours followed with cycloheximide pulse-chase assay. The results are quantified and represented as mean ± s.d. from three independent experiments (lower panel). E) MDA-MB-231 cells stably expressing control or CDK1 shRNA were transfected with indicated plasmids. Cell lysates were subjected to immunoprecipitation with anti-Flag antibody and the ubiquitination of TWIST1 was examined by western blotting. F) BT549 Cells were transfected with indicated plasmids and treated with vehicle or RO-3306 for 24 hours in the presence of MG-132 (10 μM). Cell lysates were subjected to immunoprecipitation with anti-Flag antibody and the ubiquitination of TWIST1 was examined by western blotting. G-H) MDA-MB-231 (G) and BT549 (H) cells were transfected with indicated plasmids. Western blot was performed with indicated antibodies. I) BT549 cells were transfected with indicated plasmids and treated with vehicle or RO-3306. Western blot was performed with indicated antibodies. J) Representative images of migration, invasion and mammosphere formation assay from cells described in (G). For migration and invasion assay, scale bar=200 μm; For mammosphere formation assay, scale bar=100 μm. K) Representative images of

migration, invasion and mammosphere formation of MDA-MB-231 cells. Cells stably expressing empty vector or Flag-TWIST1 were treated with RO-3306 and the assay were performed as indicated. For migration and invasion assay, scale bar = 200 um; for mammosphere formation assay, scale bar = 100 μm. L) Graphic representation of the migration ability from MDA-MB-231 cells described in (G) was examined by transwell migration assay. The results were represented as mean ± s.d. from three independent experiments; $**p < 0.01$. M) Graphic representation of the invasion capacity of MDA-MB-231 cells described in (K). The results were represented as mean ± s.d. from three independent experiments; $**p<0.01$. n,o Graphic representation of mammosphere formation assay from MDA-MB-231 cells in (G) or (K). The results were represented as mean ± s.d. from three independent experiments; $**p<0.01$. P) Graphic representation of the migration capacity of BT549 cells examined by transwell migration assay and described in (H). The results were represented as mean ± s.d. from three independent experiments; $**p < 0.01$. Q) Graphic representation of the invasion capacity of BT549 cells described in (I). The results were represented as mean ± s.d. from three independent experiments; $**p<0.01$. R-S) Graphic representation of mammosphere formation assay from BT549 cells described in (H) or (I). The results were represented as mean ± s.d. from three independent experiments; $**p<0.01$. T-W) BT549 Cells as in (H) or (I) were treated with cisplatin (T,U) or paclitaxel (V,W) and the cell survival was determined. The results were represented as mean ± s.d. from three independent experiments.

Supplementary Figure 6. CDK1-mediated phosphorylation of USP29 regulates TWIST1 protein stability and subsequent EMT and CSC self-renewal.

A) MDA-MB-231 cells stably expressing empty vector or Flag-TWIST1 were treated with vehicle or RO-3306. Cell lysates were subjected to immunoprecipitation with anti-Flag antibody and followed by western blotting with indicated antibodies. B) BT549 cells were transfected with indicated plasmids. Cell lysates were subjected to immunoprecipitation with anti-HA antibody and followed by western blotting with indicated antibodies. C-D) MDA-MB-231 (C) and BT549 (D) cells stably expressing

control or USP29 shRNAs were treated with vehicle or RO-3306 and immunoblotted with indicated antibodies. E-F) MDA-MB-231 (E) and BT549 (F) cells were transfected with empty vector, USP29 WT or the phosphorylation-deficient mutant (3A) and cycloheximide pulse-chase assay was performed. The quantified results were represented as mean ± s.d. from three independent experiments (right panel). G) MCF-7 cells were transfected with indicated plasmids and immunoblotted with indicated antibodies. H) Immunofluorescence (IF) images of E-cadherin and Vimentin in MCF-7 cells described in (G). Nuclei were immunostained using DAPI (blue). Scale bars= 25 μm. I) Representative Images of migration, invasion and ammosphere formation assay. MDA-MB-231 cells stably expressing control or USP29 shRNA were transfected with indicated plasmids and treated with vehicle or RO-3306. Scale bar=100 μm. J) Graphic representation of the invasion ability from cells described in (I). The results were represented as mean ± s.d. from three independent experiments; ** $p<0.01$. K) Graphic representation of mammosphere formation assay in (I). The results were represented as mean ± s.d. from three independent experiments; ** $p<0.01$. L) BT549 cells stably expressing USP29 shRNA were transfected with indicated plasmids and immunoblotted with indicated antibodies. M-N) Graphic representation of the migration (M) and invasion (N) capacity of cells described in (L). The results were represented as mean ± s.d. from three independent experiments; ** $p<0.01$. O-P) Graphic representation of mammosphere formation assay from cells described in (L). The results were represented as mean ± s.d. from three independent experiments; ** $p<0.01$. Q-R) Cells as in (L) were treated with cisplatin (Q) or paclitaxel (R) and cell survival was determined. The results were represented as mean ± s.d. from three independent experiments.

Supplementary Figure 7. The expression of CDK1 and USP29 is positively correlated with poor patient outcomes in breast cancer.

A-B) Positive correlation of TWIST1 expression with USP29 (B) and CDK1 (C). C-D) Survival curve evaluating the prognostic value of USP29 in TNBC patients. Survival curves for PFS (C) and DMFS (D) were generated by the Kaplan-Meier plotter. E-F)

Survival curve evaluating the prognostic value of CDK1 in breast cancer patients. Survival curves for PFS (E) and DMFS (F) generated by the Kaplan-Meier plotter.

Supplementary Figure 1

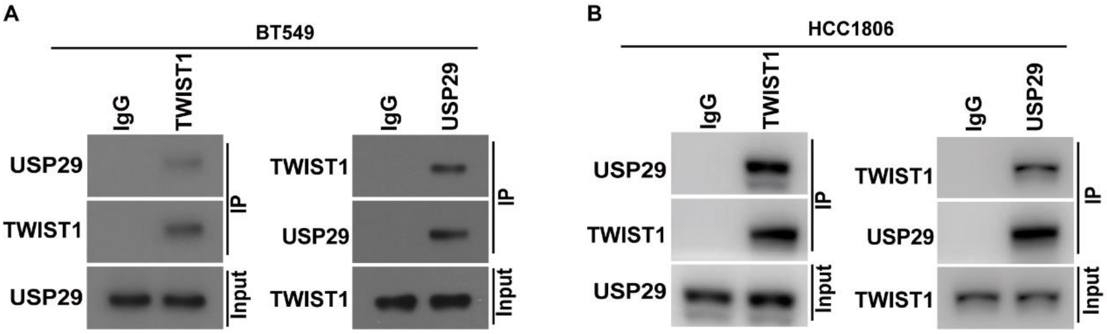

Supplementary Figure 2

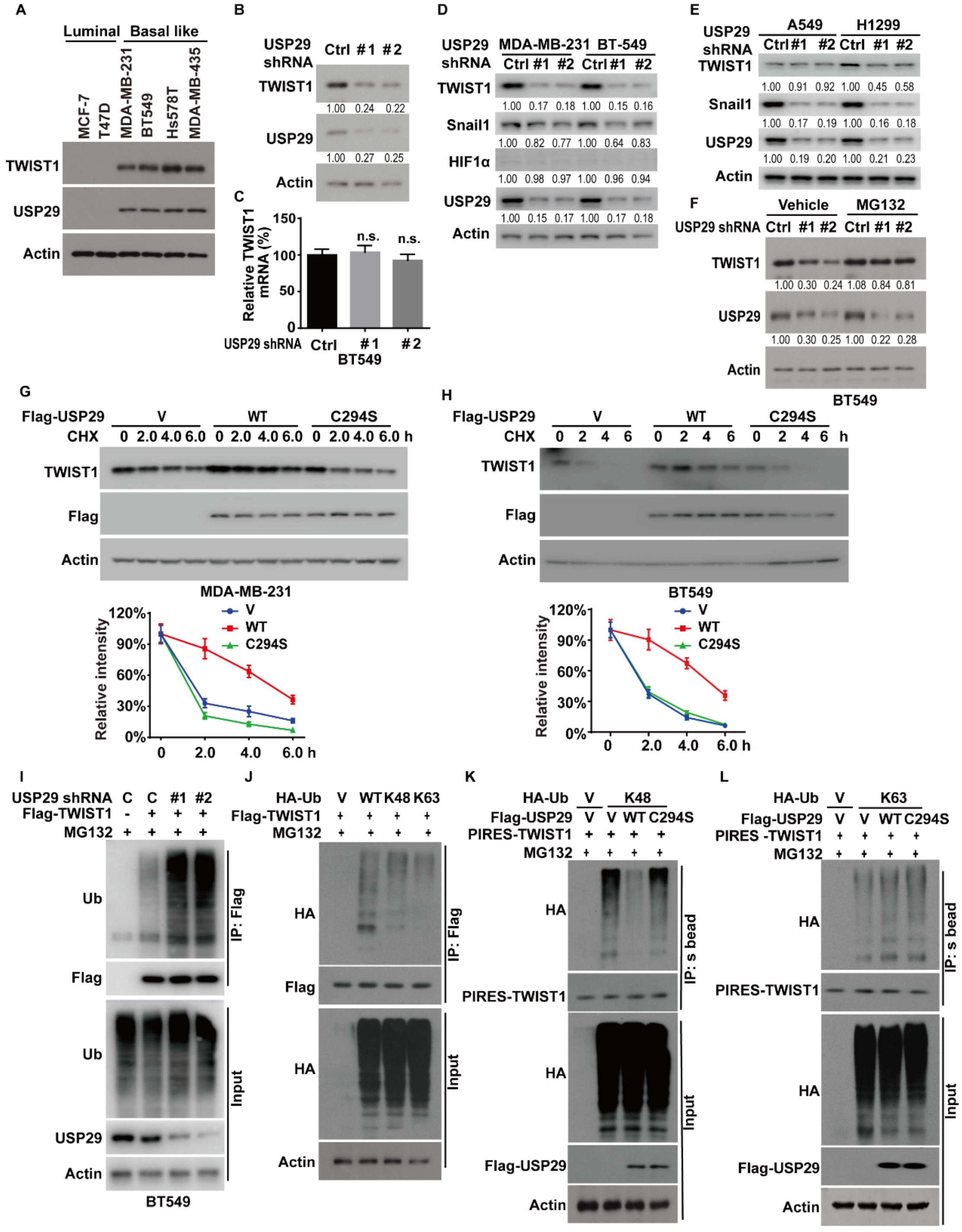

Supplementary Figure 3

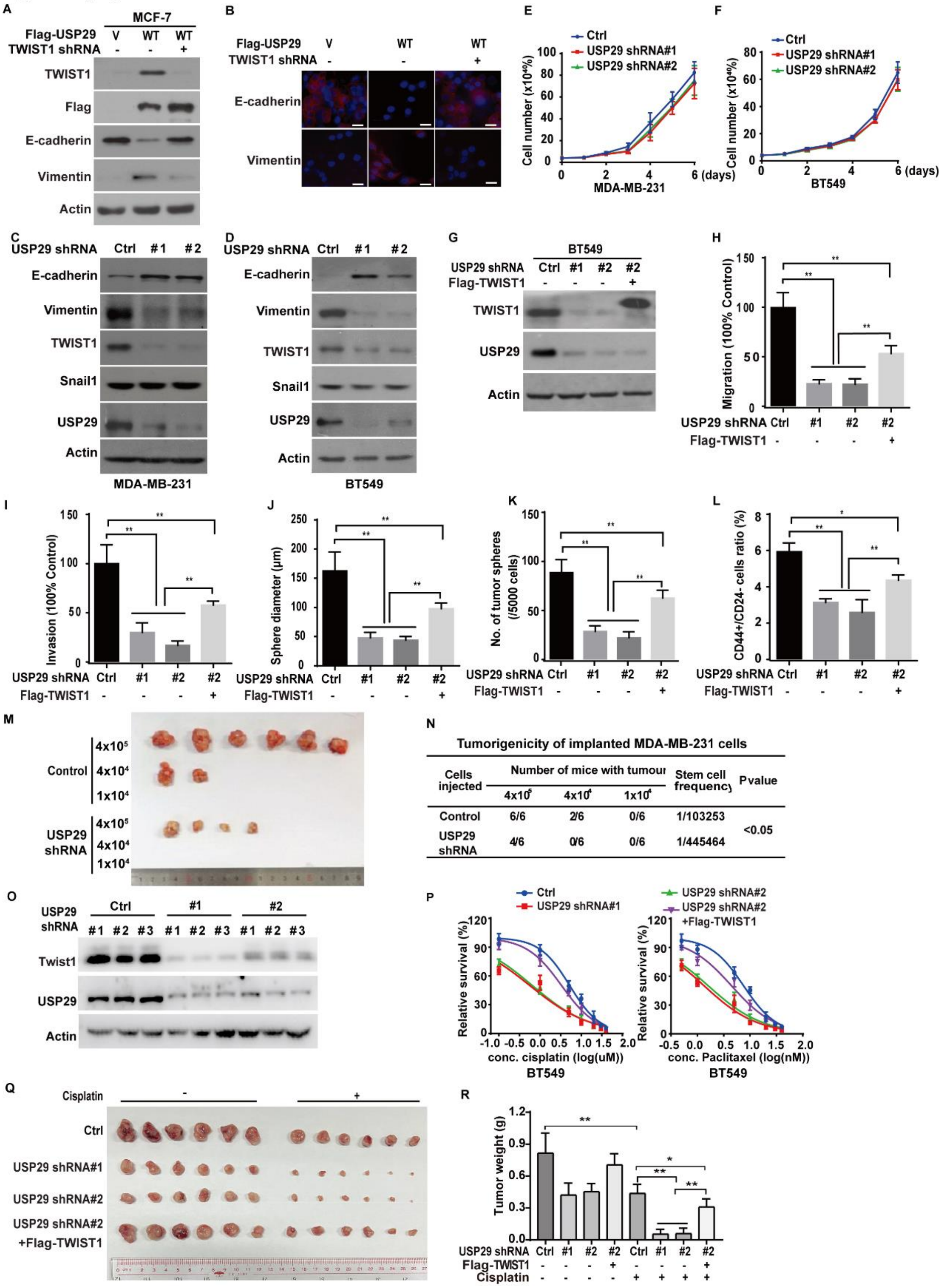

Tumorigenicity of implanted MDA-MB-231 cells

Cells injected	Number of mice with tumour 4x10⁵	4x10⁴	1x10⁴	Stem cell frequency	Pvalue
Control	6/6	2/6	0/6	1/103253	<0.05
USP29 shRNA	4/6	0/6	0/6	1/445464	

Supplementary Figure 4

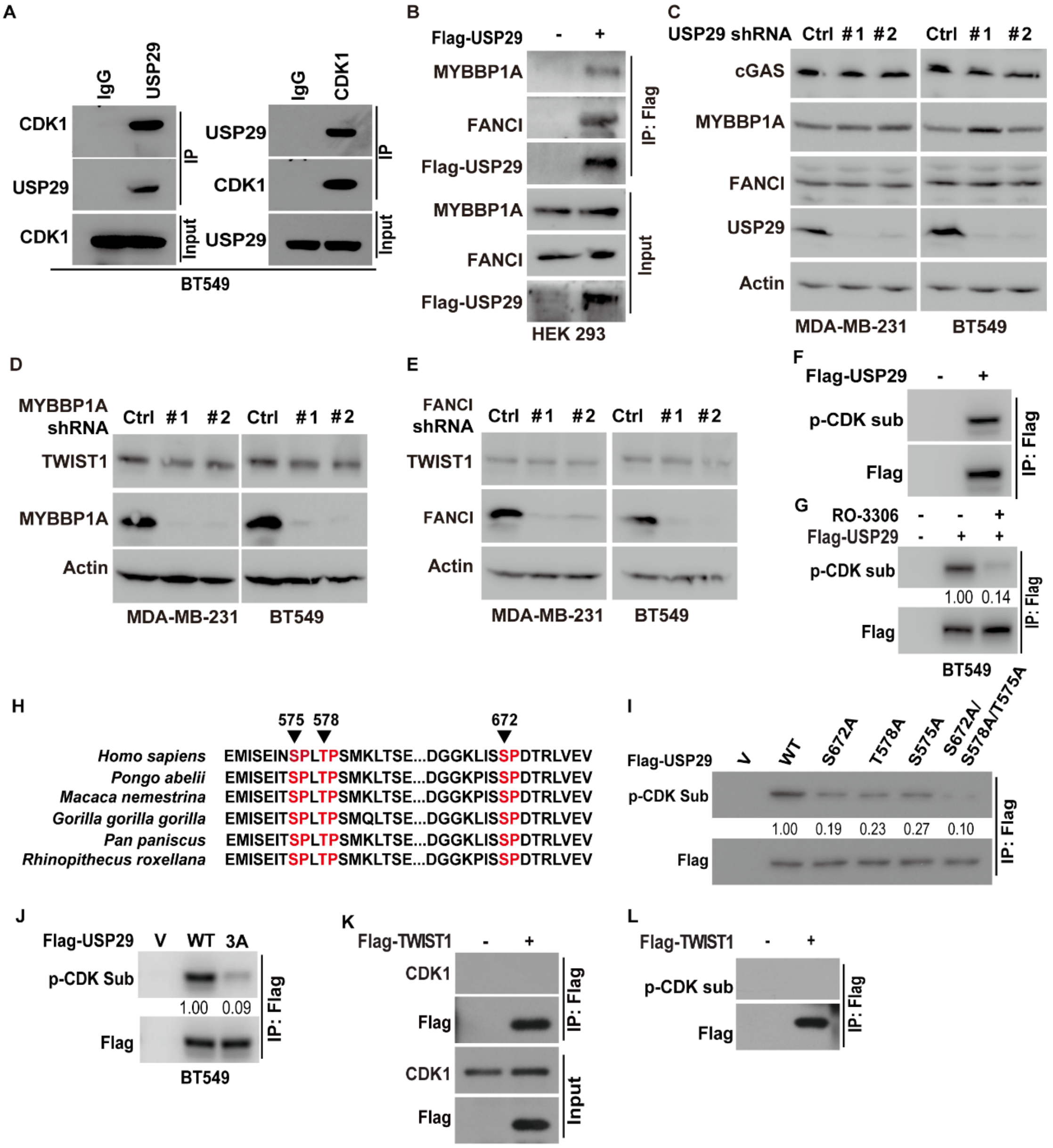

Supplementary Figure 5

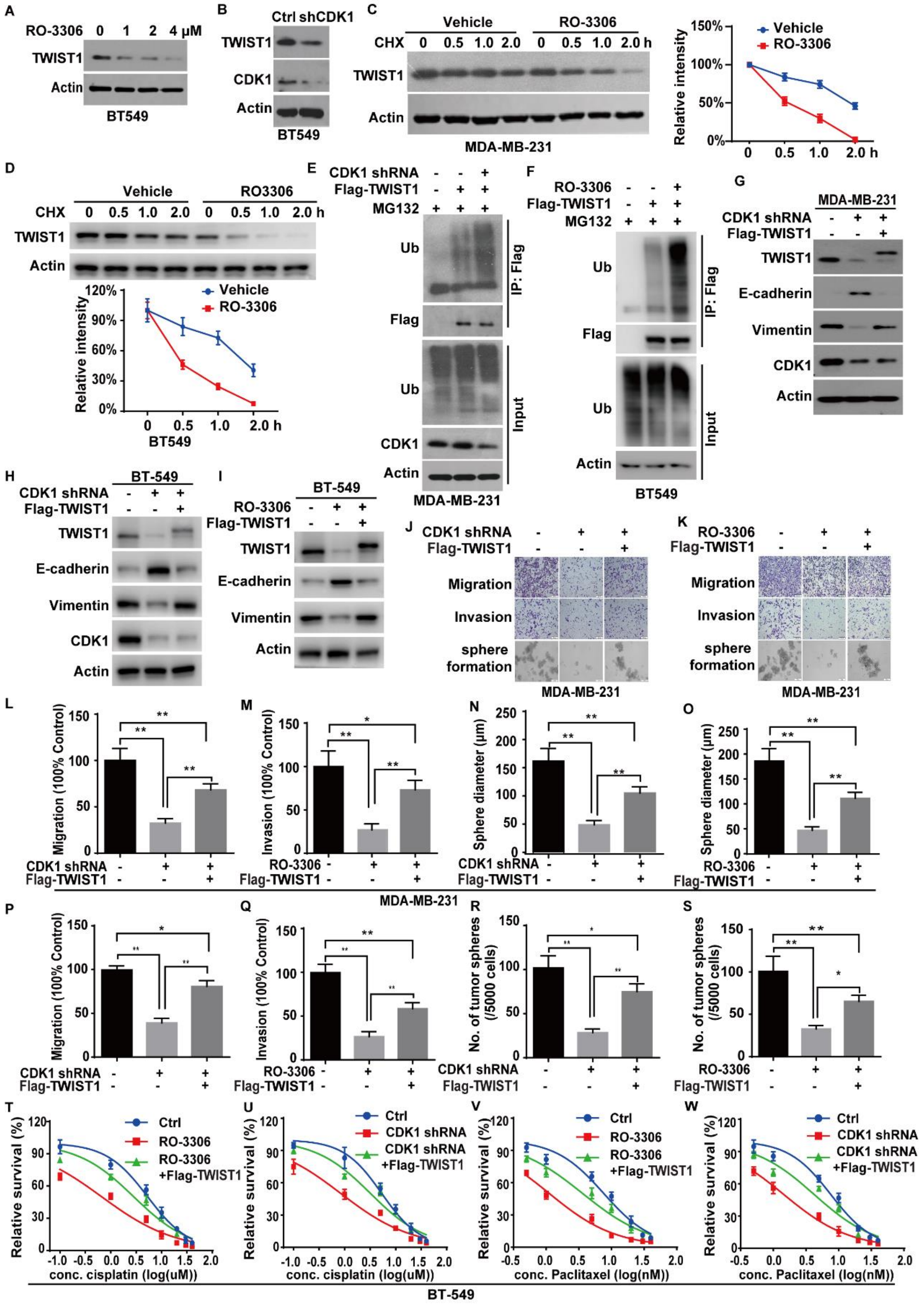

Supplementary Figure 6

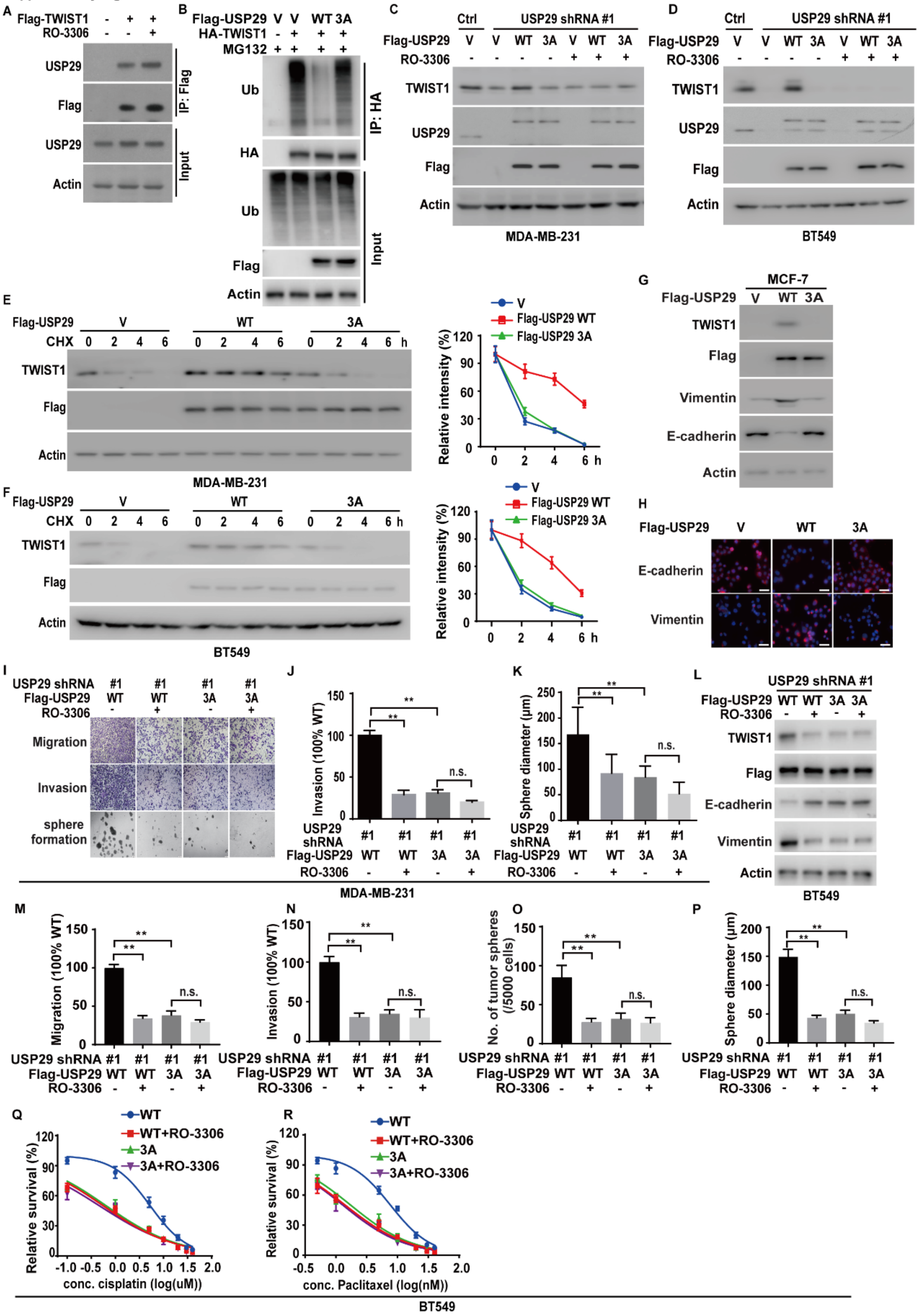

Supplementary Figure 7

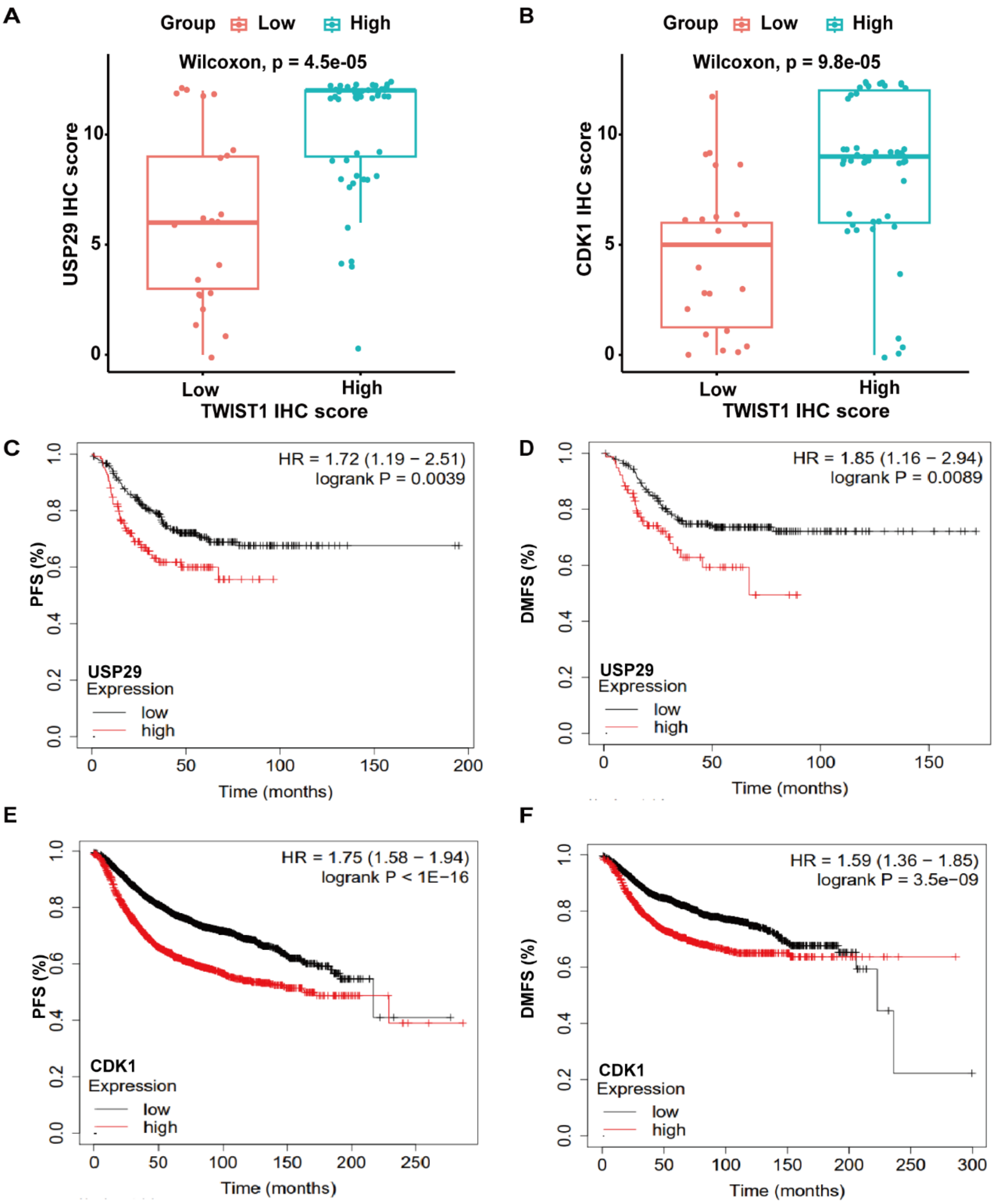

Supplement: Supplementary file 1 — Supporting information [file ADVS-10-2205873-s001.pdf]
